# The SPATA5-SPATA5L1 ATPase complex directs replisome proteostasis to ensure genome integrity

**DOI:** 10.1016/j.cell.2024.03.002

**Published:** 2024-03-29

**Authors:** Vidhya Krishnamoorthy, Martina Foglizzo, Robert L. Dilley, Angela Wu, Arindam Datta, Parul Dutta, Lisa J. Campbell, Oksana Degtjarik, Laura J. Musgrove, Antonio N. Calabrese, Elton Zeqiraj, Roger A. Greenberg

**Affiliations:** 1 Department of Cancer Biology, Penn Center for Genome Integrity, Basser Center for BRCA, Perelman School of Medicine, University of Pennsylvania, Philadelphia, PA 19104-6160; 2 Astbury Centre for Structural Molecular Biology, School of Molecular and Cellular Biology, Faculty of Biological Sciences, University of Leeds, Leeds, LS2 9JT, UK

**Keywords:** 55LCC, SPATA5, SPATA5L1, C1orf109, CINP, AAA+, ATPase, unfoldase, replication stress response, genome instability, replisome regulation/proteostasis

## Abstract

Ubiquitin-dependent unfolding of the CMG helicase by VCP/p97 is required to terminate DNA replication. Other replisome components are not processed in the same fashion, suggesting that additional mechanisms underlie replication protein turnover. Here, we identify replisome factor interactions with a protein complex composed of AAA+ ATPases SPATA5-SPATA5L1 together with heterodimeric partners C1orf109-CINP (55LCC). An integrative structural biology approach revealed a molecular architecture of SPATA5-SPATA5L1 N-terminal domains interacting with C1orf109-CINP to form a funnel-like structure above a cylindrically-shaped ATPase motor. Deficiency in the 55LCC complex elicited ubiquitin-independent proteotoxicity, replication stress, and severe chromosome instability. 55LCC showed ATPase activity that was specifically enhanced by replication fork DNA and was coupled to cysteine protease-dependent cleavage of replisome substrates in response to replication fork damage. These findings define 55LCC-mediated proteostasis as critical for replication fork progression and genome stability and provide a rationale for pathogenic variants seen in associated human neurodevelopmental disorders.

**Figure:**
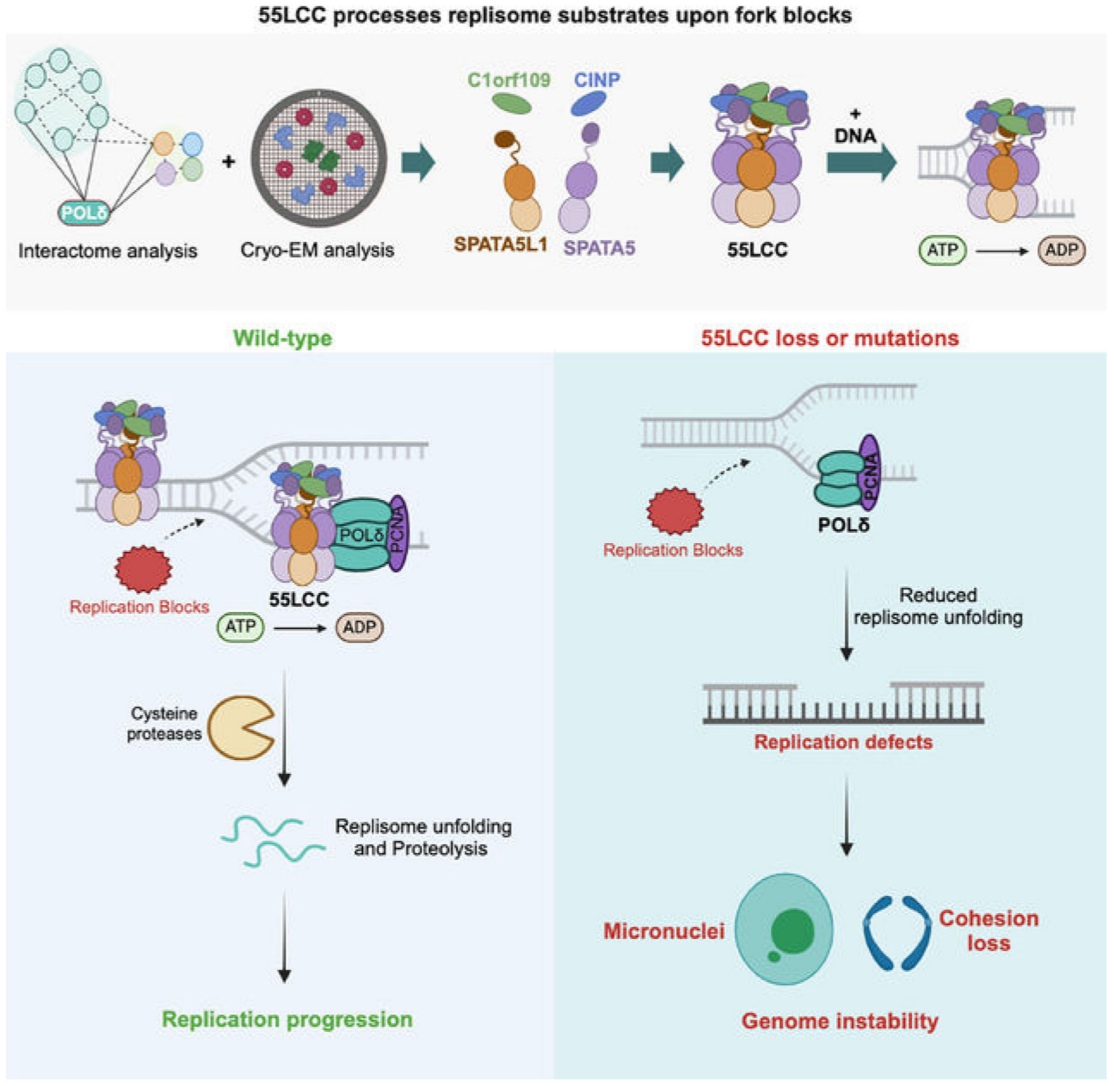

## Introduction

Cellular protein homeostasis is maintained by myriad enzymatic activities that include protein unfolding and proteolytic turnover^1^. How these processes are enacted to control DNA replication is of central importance to genome maintenance. Studies of replication termination provide key insights into replisome recycling. Upon completion of DNA synthesis, DNA replication is terminated by ubiquitylation of the replicative helicase CMG (CDC45, MCM2–7, GINS), which is then recognized and unfolded by the AAA+ ATPase p97/VCP in association with ubiquitin-interacting adaptor proteins^2–7^. Remarkably, ubiquitin-dependent unfolding and disassembly of the CMG helicase is not only required for DNA replication termination, but paradoxically, also for efficient DNA synthesis through S-phase due to a need for continuous replisome turnover and recycling for new origin firing^8^.

Lagging strand DNA synthesis is primarily executed by DNA polymerase δ (Pol δ). This four-subunit polymerase complex does not directly associate with the CMG but instead accesses replication forks by binding to the DNA clamp, PCNA (proliferating cell nuclear antigen)^9,10^. Interaction with PCNA and independence from the CMG allows Pol δ to participate in lagging strand DNA replication as well as homology-directed repair at replication forks and telomeres^11,12^. Additionally, POLD3 is a common subunit of the translesion synthesis polymerase, DNA polymerase ζ, further highlighting the expansive roles of Pol δ complex members in DNA synthesis reactions.

We posited that DNA Pol δ would be subjected to regulatory events during DNA replication, albeit distinct from those that affect the CMG. Here we identify the interaction of Pol δ and other replisome components with the AAA+ ATPases SPATA5 and SPATA5L1, which are mutated in the human neurodevelopmental syndromes EHLMRS (Epilepsy, Hearing Loss, and Mental Retardation Syndrome) and NEDHLS (Neurodevelopmental Disorder with Hearing Loss and Spasticity)^13–18^. We show that SPATA5 and SPATA5L1 associate in a DNA-binding heterohexameric complex together with heterodimeric alpha-helical proteins C1orf109 and CINP. This chromatin-associated complex, which we name 55LCC, uses ATPase activity to process replisome substrates in S-phase, facilitating their proteolytic turnover from chromatin to ensure DNA replication and mitotic fidelity. These findings define a mechanism of DNA replication proteostasis by a multi-subunit macromolecular motor that is necessary for genome stability.

## Results

### Replisome interactions with the SPATA5-SPATA5L1-C1orf109-CINP (55LCC) AAA+ ATPase complex

**Figure 1:**
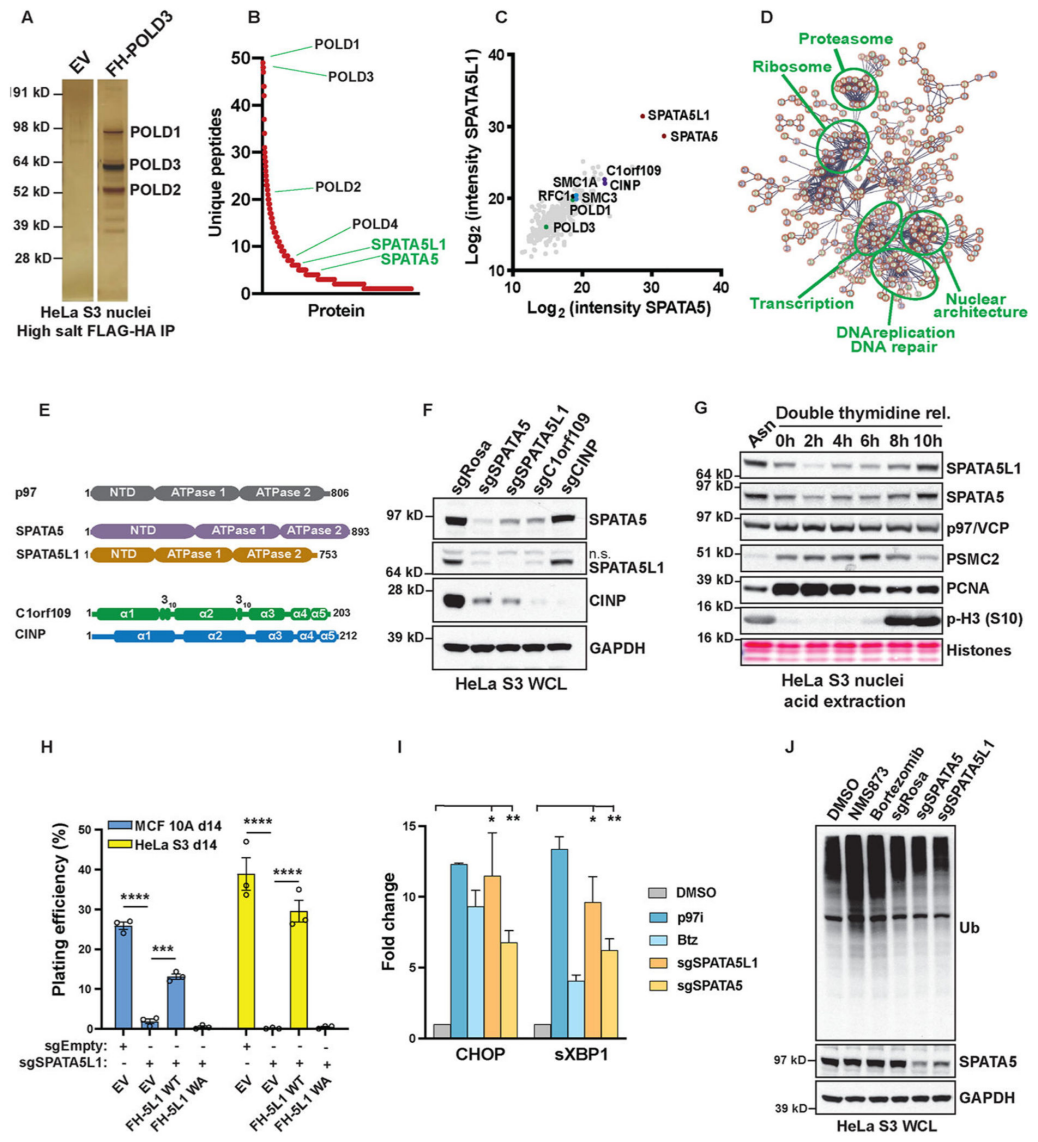
SPATA5 and SPATA5L1 form a complex with CINP and C1orf109 that interacts with the POLD3 replisome and is essential for cell viability and protein homeostasis **A.** Silver stain of FLAG-HA tandem purified samples from high-salt extracted HeLa S3 nuclei stably expressing FH-POLD3 or EV. TCA precipitated samples were analyzed by mass spectrometry. Data in Table S1. FH = FLAG-HA, EV = empty vector. **B.** Quantification of mass spectrometry unique peptide number for proteins identified in **A**. **C.** Mass spectrometry of FLAG-HA affinity purified samples from high-salt extracted HeLa S3 nuclei stably expressing FH constructs or EV with data analysis represented in **C-D**. Quantification of the intensities of the identified proteins from FH-SPATA5 and FH-SPATA5L1. Data in Table S1. **D.** STRING interaction network of proteins from mass spectrometry of FH-SPATA5L1 purified sample. **E.** Schematics depicting the domain organization of indicated proteins. NTD = N-terminal domain. **F.** Western blot of whole cell lysates (WCL) from HeLa S3 treated with indicated CRISPR guides (sg) or control (sgRosa) for 8 days. n.s. indicates non-specific bands. **G.** Western blot of acid extracted nuclei from asynchronous (Asn) and double thymidine block released samples of HeLa S3. Histones visualized with Ponceau stain. **H.** Quantification of plating efficiency of MCF 10A and HeLa S3 stably expressing FHS-PATA5L1 constructs or EV treated with indicated CRISPR guides for 14 days. Data are mean α SEM; n=3 from 1 biological replicate. *p*-values determined by unpaired Student’s *t*-test. WT = wild type, WA = Walker A double mutant. **I**. RealTime-qPCR of integrated stress response genes CHOP and spliced XBP1 of HeLa S3 cells treated with indicated inhibitors for 4 hours (h) or CRISPR guides for 10 days. n=3 biological replicates; n=2 for Bortezomib (Btz) and sgSPATA5 samples. *p*-values determined by unpaired Student’s *t*-test. **J**. Western blot of WCL from HeLa S3 treated with inhibitors for 4 h or the indicated CRISPR guides for 8 days.

We performed tandem affinity purification of POLD3 from HeLa S3 nuclear extracts, which revealed a near stoichiometric interaction with Pol δ subunits POLD1 and POLD2 as well as interactions with Pol ζ constituents REV7, REV3L, and REV1 (Figures 1A and B, Table S1). Mass spectrometry results also revealed numerous peptides in the POLD3 purification from the poorly characterized AAA+ ATPases SPATA5 and SPATA5L1. Flag-immunoprecipitation (IP) experiments confirmed the interaction between ectopically expressed POLD3 and both SPATA5 and SPATA5L1 (Figure S1A) and reverse co-immunoprecipitation (co-IP) identified the interactions of FLAG-SPATA5L1 with replisome components POLD3 and RFC1 on chromatin (Figure S1B). Tandem affinity purification of SPATA5 and SPATA5L1 identified association with several DNA replication factors, including POLD3, POLD1, RFC1, and the cohesin subunits SMC1 and SMC3 (Figures 1C and D, Table S1). SPATA5 and SPATA5L1 also interacted with protein complexes involved in various cellular functions in the nucleus and cytoplasm (Figures 1D and S1C, Table S1), including translation, protein degradation, and nuclear architecture, suggestive of a broad role in protein homeostasis. CINP and C1orf109 were among the highest confidence hits in the mass spectra from both SPATA5 and SPATA5L1 purifications (Figure 1C, Table S1). In accordance, tandem affinity purification of C1orf109 and CINP revealed SPATA5 and SPATA5L1 as top interactors and reciprocal co-IPs confirmed the interaction of all four subunits (Figures S1D–F, Table S1). Additionally, SPATA5, SPATA5L1, C1orf109, and CINP showed co-dependency in the Cancer Dependency Map^19^.

SPATA5 and SPATA5L1 belong to the CDC48 family of type II AAA+ ATPases, which contain two ATPase domains separated by an intervening sequence and an N-terminal region that typically is used to specify protein-protein interactions (Figures 1E, S1G, and S2A)^20^. Among other members of the CDC48 family, SPATA5 and SPATA5L1 show the highest degree of structure and sequence similarity to each other and to p97 (Figure S2). While little is known about C1orf109^21,22^, CINP was shown to interact with DNA replication factors and was required for genome stability^23–25^. CRISPR-mediated deletion showed co-dependency for stability of SPATA5, SPATA5L1, and C1orf109 (Figures 1F and S1H). CINP required expression of all other subunits for its stability but loss of CINP did not substantially affect levels of the other three members (Figure 1F). These data indicate that SPATA5, SPATA5L1, C1orf109, and CINP exist as a stable complex (55LCC) in cells.

Interaction between the 55LCC complex and replisome components suggests a chromatin-associated function. Synchronization experiments using double thymidine block showed chromatin association of SPATA5 and SPATA5L1 that peaked in G2/M and was reduced in S-phase (Figure 1G), in agreement with a report that CINP displays reduced chromatin association in S-phase^25^.

### The 55LCC complex is required for ubiquitin-independent proteostasis

All four 55LCC components were required for viability across several cell lines, consistent with their designation as pan-essential genes (Figures 1H and S1I–K)^19^. p97 is also essential for cellular viability and mediates protein quality control by coupling ubiquitin- and ATPase-dependent protein unfolding through its central pore to proteasome-mediated degradation^26–29^. The SPATA5 and SPATA5L1 interactomes demonstrated association with many different nuclear and cytoplasmic protein complexes (Figures 1D and S1C, Table S1), suggesting that they contribute to global proteostasis. Like p97 or proteasome inhibition, loss of 55LCC complex integrity elicited an unfolded protein response^30^, characterized by increased expression of CHOP (CAAT/enhancer binding protein) and spliced XBP1 (X-box binding protein 1) (Figure 1I). However, SPATA5 and SPATA5L1 deficiency did not lead to an accumulation of high molecular weight ubiquitylated proteins (Figure 1J), suggesting that 55LCC complex does not act on ubiquitylated substrates to facilitate proteostasis, differentiating it from p97.

### The 55LCC complex is stabilized by ATP and binds DNA

**Figure 2:**
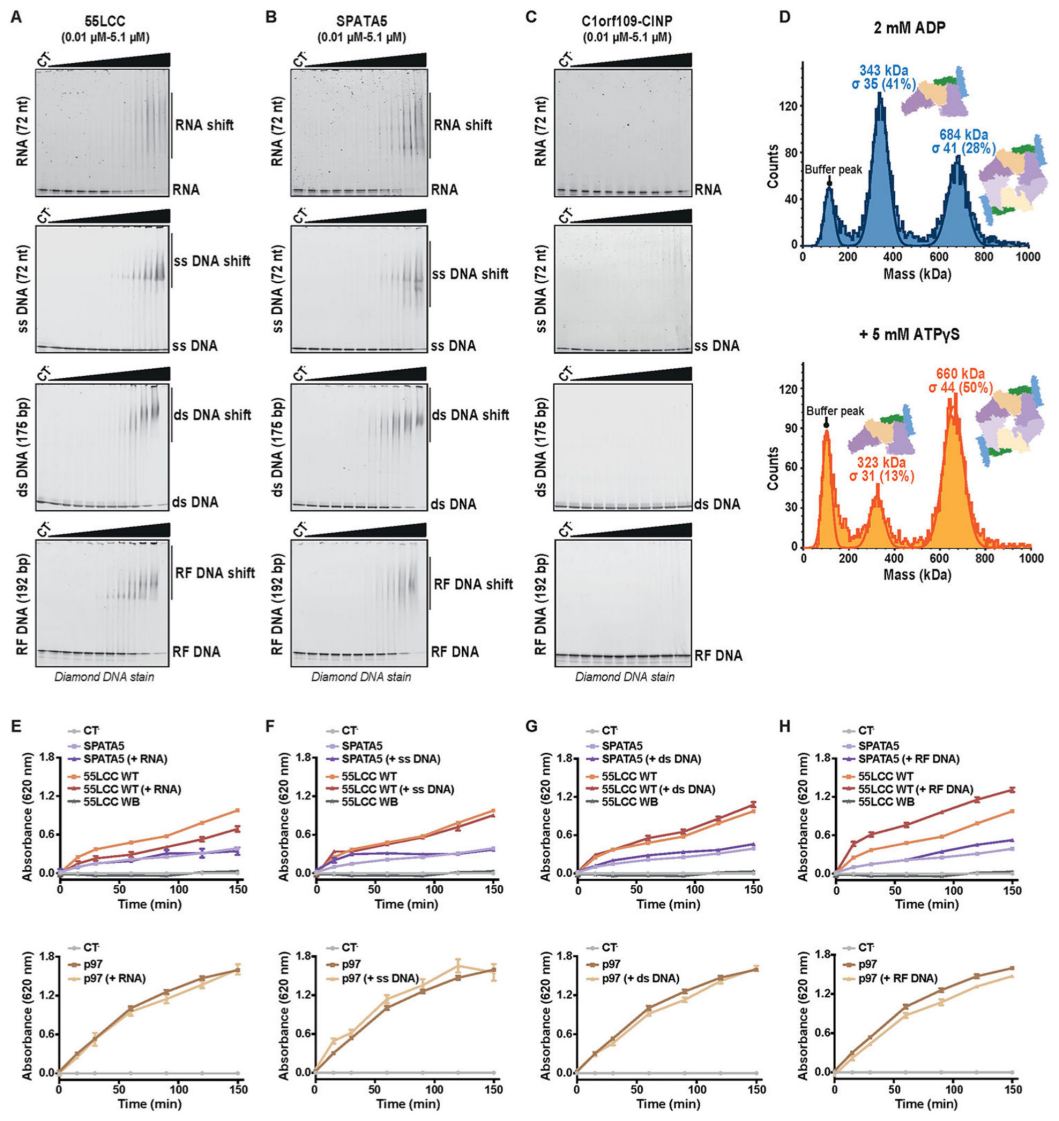
The SPATA5-SPATA5L1-C1orf109-CINP (55LCC) complex is a DNA-binding ATPase **A.-C**. Electrophoretic mobility shift assays (EMSAs) testing 55LCC (**A**), SPATA5 (**B**), and C1orf109-CINP (**C**) binding to RNA and to single-stranded (ss), double-stranded (ds) and replication fork (RF) DNA variants (2.2 ng/μl each corresponding to 84 nM, 17 nM and 16 nM of ss, ds and RF DNA respectively, and to 86 nM of RNA) at increasing protein concentrations (from 0.01 μM to 5.1 μM in 2-fold dilution steps). This resulted in protein:DNA ratio ranges of ~0.1–61, ~0.6–300 and ~0.6–319 for ss, ds and RF DNA variants, and of ~0.1–59 for RNA. Gels were stained with Diamond^™^ nucleic acid dye. Data are representative of two independent experiments. CT^−^ = negative control. **D**. Mass distribution of 55LCC measured by mass photometry in the presence of 2 mM ADP (*top*), and after addition of 5 mM ATP***γ***S (*bottom*). Observed masses for each peak are indicated. The percentage of particles contributing to each peak is shown in brackets (see Table S2 for details). Schematics depicting indicated complexes and presumed sub-complexes consistent with the measured Mw are shown; SPATA5 and SPATA5L1 subunits are colored in shades of purple and orange respectively, while C1orf109-CINP are colored green and dark blue. Data are representative of two independent experiments carried out in technical duplicates. **E.-H**. ATPase activities of SPATA5, 55LCC variants (at 1 μM each; *top*) and p97 (at 0.1 μM; *bottom*) were determined in the absence or presence of RNA (**E**), and of ss (**F**), ds (**G**) and RF (**H**) DNA types (at 2 μM each). Results are the average of three independent experiments carried out in technical duplicates. Mean with SEM was used to plot the data; error bars shorter than the height of the symbol are not visible. CT^−^ = negative control, WT = wild-type, WB = Walker B double mutant.

Given the interaction of SPATA5-SPATA5L1 with replication factors, we determined whether the *in vitro* reconstituted 55LCC complex or any of its constituents (Figure S3A) binds RNA or DNA using an electrophoretic mobility shift assay (EMSA). Both SPATA5 and 55LCC interacted with RNA and all DNA forms, although they displayed a preference for double-stranded (ds) and replication fork (RF) DNA over RNA and single-stranded (ss) DNA (Figures 2A and B). Of note, 55LCC interaction with RNA was the weakest among all nucleic acids tested. By contrast, we observed no shift for the C1orf109-CINP sub-complex in the presence of any nucleic acids (Figure 2C). Collectively, these data show that the 55LCC complex specifically binds DNA and suggest that these interactions are largely mediated by SPATA5. Notably, we could not purify SPATA5L1 alone, suggesting that interactions with SPATA5 and C1orf109-CINP are required for stable SPATA5L1 expression and purification *in vitro*.

To measure the molecular weight of 55LCC we performed mass photometry measurements under different nucleotide conditions. In the presence of ADP we detected an oligomeric assembly with a mass consistent with a SPATA5-SPATA5L1 heterohexamer plus two copies of C1orf109-CINP complex (Figure 2D, *top*, Table S2). A more prominent and lower molecular weight (Mw) peak corresponding to a dissociated sub-complex was also apparent under these conditions (Figure 2D, *top*, Table S2). Addition of a non-hydrolyzable ATP analogue, ATP***γ***S, promoted a nearly two-fold increase in the 55LCC peak with a concomitant reduction of the dissociated species (Figure 2D, *bottom*, Table S2). These analyses indicate that ATP binding enhances the stability of 55LCC thus differentiating SPATA5 and SPATA5L1 from p97, whose homohexameric structure can stably assemble under any nucleotide-bound conditions^31,32^.

### The ATPase activity of 55LCC is stimulated by DNA binding

To investigate the ATPase activities of SPATA5 and of the reconstituted 55LCC complex, we used an *in vitro* colorimetric assay measuring inorganic phosphate release after ATP hydrolysis. We performed these experiments in the absence and presence of different DNA or RNA species to assess their ability to stimulate SPATA5 and 55LCC ATP hydrolysis. SPATA5 alone displayed limited ATPase activity while 55LCC wild-type (WT) showed a 3.6-fold increase in ATP hydrolysis compared to SPATA5 alone (Figures 2E–H, *top*). We observed a further enhancement in 55LCC ATPase activity following addition of RF DNA, but not of RNA or any other DNA types, to the reactions (Figures 2E–H, *top*). Notably, RNA caused a reduction in 55LCC-mediated ATP hydrolysis but had no effect on SPATA5 ATPase activity (Figure 2E, *top*). By contrast, VCP/p97 activity was not affected by addition of RNA or of any DNA variants (Figures 2E–H, *bottom*, and S3A). As expected, 55LCC activity was dependent on the conserved Walker B (WB) motifs in SPATA5 and SPATA5L1 as mutations of these residues completely abolished ATP hydrolysis (Figures 2E–H, *top*, and S2B). These analyses provide evidence of a recombinantly produced 55LCC complex with ATPase activity, and further suggest that DNA structures can specifically enhance 55LCC ATP hydrolysis.

### Cryo-EM structure of the 55LCC complex reveals an unconventional ATPase motor architecture

**Figure 3:**
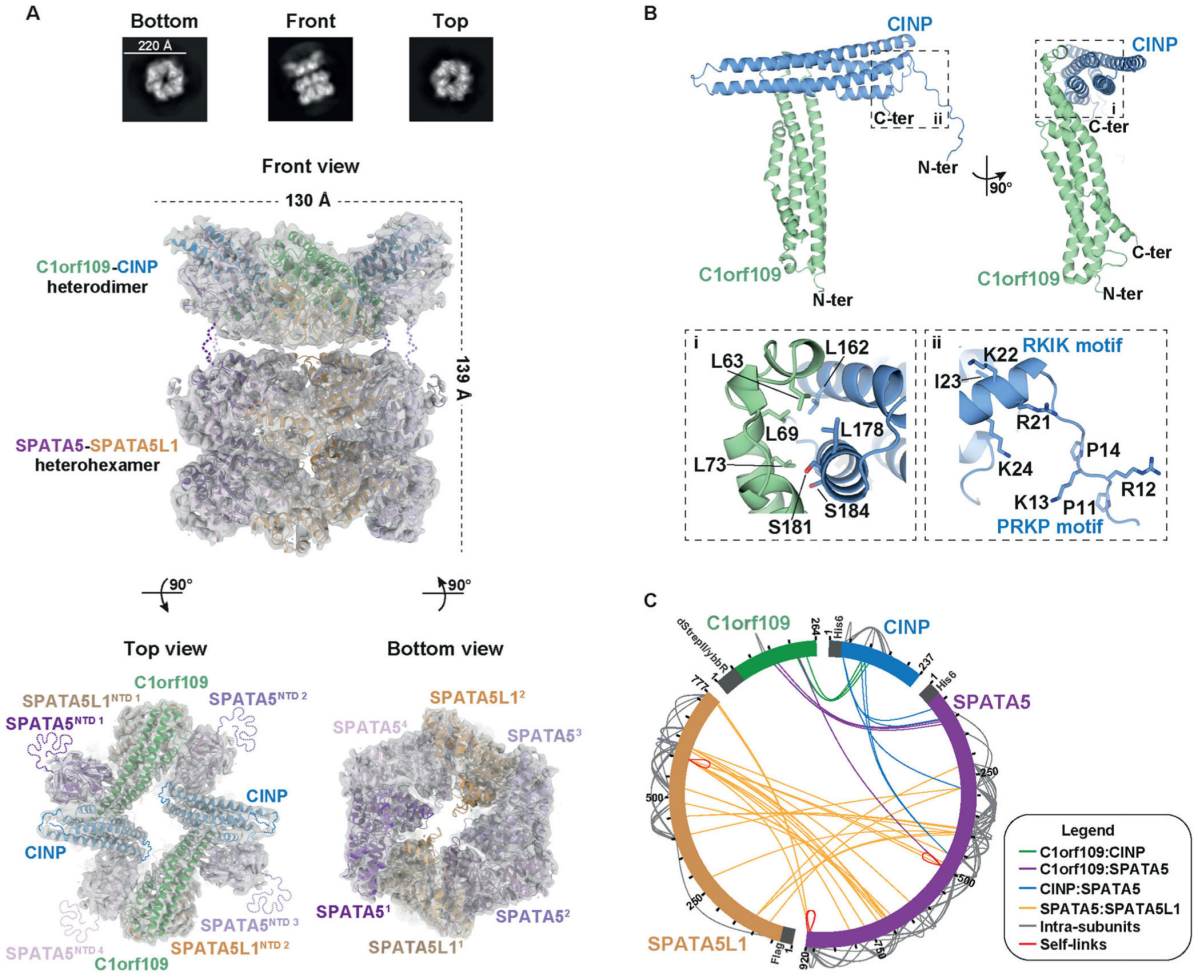
Analyses of 55LCC and C1orf109-CINP complexes **A.** Representative 2D class averages from cryo-EM analysis of 55LCC. Data are representative of 165,778 selected particles (*top*). Cryo-EM composite map of 55LCC at contour level of 0.131 is depicted in transparent surface, with front, top and bottom views indicated. Cartoon models of the C1orf109-CINP heterodimer and the SPATA5-SPATA5L1 heterohexamer were rigid-body fitted in the cryo-EM density and colored as in Figure 2D. Unresolved linker regions connecting the 55LCC lid and ATPase domains, missing loops within each CINP promoter or unstructured regions in the SPATA5 N-termini are indicated with dashed lines; superscript numbers refer to the SPATA5 and SPATA5L1 subunits (*bottom*). See Figures S3B–H and Table S3a for details. NTD = N-terminal domain. **B.** Cartoon model of the C1orf109-CINP complex predicted by AlphaFold-Multimer, with C1orf109 and CINP colored in green and dark blue respectively; dashed black rectangles highlight regions analyzed by mutagenesis (*top*). Close-up views and structural details of the predicted interacting residues at the C1orf109-CINP interface (panel i), and of CINP RKIK and PRKP motifs (panel ii). Residues are labeled and shown as sticks (*bottom*). **C.** Chemical cross-linking and mass spectrometry analyses of the reconstituted 55LCC complex. Purification and labeling tags are indicated and colored gray. Data are representative of a single experiment measured in duplicate.

To understand the assembly of 55LCC, we performed single particle cryo-EM analyses of the *in vitro* reconstituted complex. We noticed severe particle preferential orientation, which we improved by using fluorinated octyl maltoside for grid preparation and that allowed us to achieve an overall resolution of 4.5 Å (Figures S3B–D, Table S3a). Subsequent image processing and focused masking of the top and bottom portions of the map improved the quality of these regions and allowed generation of a final composite map with local resolutions of 4.02 Å and 4.52 Å respectively (Figures 3A, S3B and E–G, Table S3a). We could rigid-body fit two copies of C1orf109-CINP facing each other in a diamond-shaped arrangement sitting on the top of the 55LCC composite map, with both CINP molecules directed towards the ATPase pore (Figure 3A). Notably, the individual CINP and C1orf109 structures observed in the 55LCC map were nearly identical to the structures solved by X-ray crystallography and predicted by AlphaFold 2 (Figures 3A, S3A, and S4A, Table S3b)^33,34^. The cryo-EM structure adopted by the C1orf109-CINP sub-complex also closely matched the T-like structure predicted by AlphaFold-Multimer^35^ (Figures 3A and B, and S4B; r.m.s.d. = 2.94 Å). In agreement with these observations, recombinantly purified CINP and C1orf109 behaved as monomers in solution while the C1orf109-CINP complex formed a stable heterodimer with a 1:1 stoichiometry and a Mw of 49.7 kDa when analyzed by native mass spectrometry (Table S2). We next focused our attention on six unassigned densities located in the top region of the 55LCC map, four adopting a vertical orientation surrounding the C1orf109-CINP dimers and two positioned horizontally underneath C1orf109 alpha 1 (α1), α2 and α3 helices (Figure 3A). The overall map resolution in these regions was sufficient to discriminate between the SPATA5 and SPATA5L1 N-terminal domains (NTDs) and permitted positioning of four SPATA5 NTD models in the densities surrounding the C1orf109-CINP protomers and of two SPATA5L1 NTDs underneath each C1orf109 molecule (Figures 3A and S3H). Guided by partially visible linker loops connecting the SPATA5-SPATA5L1 NTDs to their corresponding ATPase units, we fitted four and two copies of the SPATA5 and SPATA5L1 ATPase domains in the bottom part of the cryo-EM density (Figure 3A).

The structural model suggests a 4:2:2:2 stoichiometry for the SPATA5, SPATA5L1, C1orf109 and CINP subunits, forming a full 55LCC complex with an architecture not previously observed for other heterohexameric ATPases. To independently obtain an interaction map between the subunits, we performed chemical cross-linking mass spectrometry analysis (XL-MS) of 55LCC (Figure 3C). We used the XL-MS-cleavable cross-linker disuccinimidyl dibutyric urea (DSBU), which has a spacer length of 12.5 Å and a reachable Cα-Cα distance between residues of ~32 Å^36^. We identified intramolecular cross-links within each subunit and intermolecular cross-links between C1orf109:CINP, C1orf109:SPATA5, CINP:SPATA5, and SPATA5:SPATA5L1 (Figure 3C), suggesting a tightly packed and interdependent molecular assembly. Mapping of the cross-link sites in our cryo-EM model indicated that C1orf109-CINP association with the SPATA5-SPATA5L1 heterohexamer predominantly occurs via the N-terminal and ATPase 1 domains of SPATA5, and it is further stabilized by secondary structural elements in SPATA5, SPATA5L1 and C1orf109 as well as via direct contacts between SPATA5 K196 and SPATA5L1 S88 (Figures 3C and S3I). These cross-links are consistent with our cryo-EM map and model, and further support the arrangement of the SPATA5 and SPATA5L1 NTDs (Figure 3A). Interestingly, we observed no cross-links between the C1orf109-CINP sub-complex and SPATA5L1 (Figure 3C). This is consistent with the SPATA5L1 NTDs being located 40–50 Å away from each CINP protomer, thus exceeding the length of the cross-linker, and with no lysines available for cross-linking at the SPATA5L1:C1orf109 interface.

We next sought to validate the arrangement of the C1orf109-CINP heterodimer and SPATA5-SPATA5L1 heterohexamer within 55LCC using cell-based assays. We introduced mutations in interacting residues in both C1orf109 and CINP (Figures 3B, S4C and D), and tested WT and mutant proteins in co-IP experiments. Consistent with earlier results (Figure S1D), WT C1orf109 formed a stable complex with CINP and the ATPases SPATA5-SPATA5L1 (Figure S4E). Arginine or alanine mutations of C1orf109 residues L63, L69, and L73 disrupted association with CINP but did not affect SPATA5-SPATA5L1 interactions (Figures 3B, S4C and E). Similarly, corresponding mutations at the interacting CINP residues L162, S181, and S184 impaired CINP binding to SPATA5 and SPATA5L1. Conversely, mutation of the more distal residue L178, or the conserved PRKP and RKIK motifs located at the N-terminus of CINP, did not affect complex formation (Figures 3B, S4D and F). Additional co-IP experiments showed that the SPATA5 and SPATA5L1 NTDs are necessary for SPATA5-SPATA5L1 association with each other and with CINP (Figures S4G–I). By contrast, mutations in the SPATA5 ATPase domains affecting nucleotide binding and pore loop residues had little or no effect on interactions with SPATA5L1 and CINP (Figure S4H), thus showing that association between SPATA5-SPATA5L1 and C1orf109-CINP does not rely on the core ATPase units. These findings and the numerous cross-links between SPATA5 and SPATA5L1 (Figure 3C) highlight the importance of the SPATA5-SPATA5L1 NTDs in connecting the ATPase heterohexamer with C1orf109-CINP, as visualized in the cryo-EM model (Figure 3A). Importantly, cellular reconstitution experiments with CRISPR-resistant cDNA of 55LCC point mutants revealed that nucleotide binding (Walker A residues), ATPase activity (Walker B residues), and putative unfoldase activity (aromatic pore loop residues)^37–39^ are necessary to support cell viability (Figures S4J–M).

### Structural analyses of patient mutations on 55LCC structure and activity

**Figure 4:**
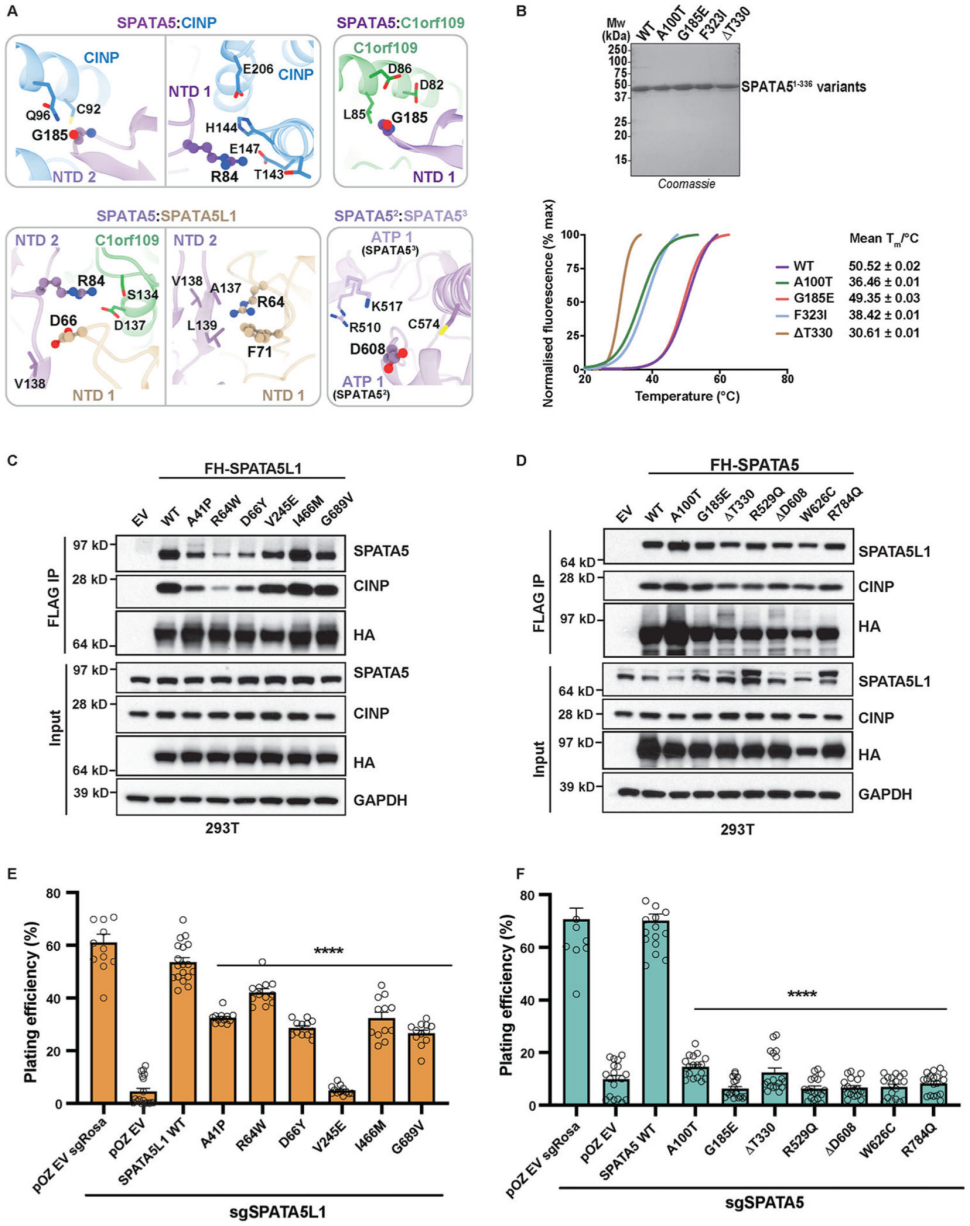
Analysis of patient mutations in SPATA5 and SPATA5L1 **A.** Close-up views and structural details of SPATA5 and SPATA5L1 residues mutated in neurodevelopmental syndromes. 55LCC subunits are shown in cartoon representation and colored as in Figure 2D. Mutated residues are labeled and shown as ball & sticks, while surrounding amino acids are indicated and shown as sticks. Superscript numbers refer to the SPATA5 subunits. See Figure S2B, Table S4, and Movie S1 for details. NTD = N-terminal domain, ATP 1 = ATPase 1 domain. **B.** Coomassie-stained SDS-PAGE analysis of purified SPATA5^1–336^ WT and disease mutants. Data are representative of a single experiment (*top*). Differential scanning fluorimetry analysis of SPATA5^1–336^ WT and disease variants. All proteins were assayed at 4 μM, and the calculated mean melting temperature (T_m_) values for each are shown. Results are the average of three independent experiments carried out in technical duplicates. Mean with SEM was used to calculate the T_m_ values for each protein (*bottom*). WT = wild type. **C.** Western blot of FLAG IP and input samples from 293T transiently expressing FH-SPATA5L1 patient mutations or EV. n=2 biological replicates. **D.** Western blot of FLAG IP and input samples from 293T transiently expressing FH-SPATA5 patient mutations or EV. n=2 biological replicates. **E.** Quantification of plating efficiency of HeLa S3 stably expressing FH-SPATA5L1 patient mutants or EV treated with indicated CRISPR guides for 14 days. n=12 from 2 biological replicates. SPATA5L1 WT and pOZ EV sgSPATA5L1 data are from n=18 from 3 biological replicates. Data are presented as mean ± SEM. *p*-values determined by one-way ANOVA with Tukey’s multiple comparison test. **F.** Quantification of plating efficiency of HeLa S3 stably expressing FH-SPATA5 patient mutants or EV treated with indicated CRISPR guides for 14 days. n=18 from 3 biological replicates. pOZ EV sgRosa data are from n=12 from 2 biological replicates. Data are presented as mean ± SEM. *p*-values determined by one-way ANOVA with Tukey’s multiple comparison test.

Germline mutations in SPATA5 and SPATA5L1 have been linked to a spectrum of human neurodevelopmental syndromes^13–18^. We used a structure guided approach to predict how disease associated mutations affect 55LCC function. Most of the mutated residues either provide structural stability and ensure correct folding of SPATA5 and SPATA5L1, or mediate protein-protein interactions (Figures 4A and S2B, Table S4, Movie S1), suggesting that these variations cause instability of the heterohexamer or complex dissociation. Furthermore, additional mutations are located in the SPATA5 and SPATA5L1 ATPase domains that bind nucleotides or are involved in substrate threading (Figures 4A and S2B, Table S4), suggesting that these mutations would likely affect 55LCC enzymatic functions. Our analyses provide an understanding of how mutations within this molecular motor may contribute to disease development.

To characterize the impact of these disease mutants on the stability of the SPATA5 NTD *in vitro*, we performed differential scanning fluorimetry. Comparison of purified WT and disease variants (Figure 4B, *top*) revealed that the G185E mutant had no effect on SPATA5 stability relative to WT protein, consistent with this residue mediating protein-protein interactions (Figure 4B, *bottom*, Table S4). By contrast, the A100T, F323I and ΔT330 mutants remarkably reduced SPATA5 melting temperature by 12–20°C, indicating that these mutations dramatically affect SPATA5 stability (Figure 4B, *bottom*, Table S4, Movie S1). This is consistent with these residues contributing to the overall fold of the SPATA5 NTD domain (Movie S1). Concomitant FLAG-immunoprecipitation experiments revealed that SPATA5L1 N-terminal patient mutants A41P, R64W, and D66Y had reduced interaction with other 55LCC members, while the ATPase domain patient mutations did not affect complex interactions (Figure 4C). SPATA5 patient mutants retained interaction with the rest of 55LCC complex proteins, with some mutations such as ΔT330 and ΔD608 displaying slightly decreased association with SPATA5L1 and CINP (Figure 4D). Interestingly, all SPATA5 patient mutants and the SPATA5L1 ATPase mutant V245E were severely compromised in cell viability in rescue experiments with CRISPR resistant cDNA of the mutants (Figures 4E and F). Furthermore, SPATA5L1 N-terminal and ATPase domain patient mutants displayed only partial reduction in colony formation (Figure 4E), suggesting that these mutations might be hypomorphic.

### The 55LCC complex is required for replication fork progression, sister chromatid cohesion, and chromosome stability

**Figure 5:**
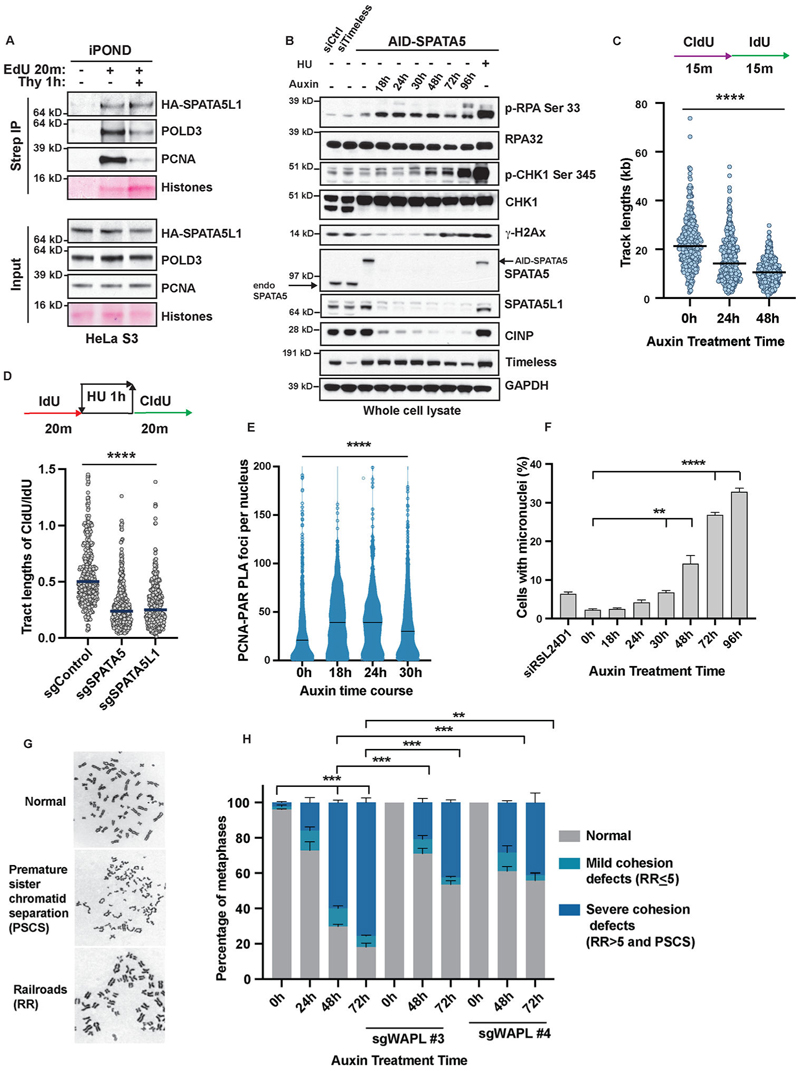
55LCC is required for DNA replication and genome stability **A.** Western blot of Streptavidin IP and input of iPOND experiment from HeLa S3 stably expressing FH-SPATA5L1 treated −/+ EdU and −/+ Thymidine chase (Thy) for indicated times. n=2 biological replicates. **B.** Western blot of WCL from HeLa S3 AID-SPATA5 cells treated with auxin for indicated times. HeLa S3 cells treated with indicated siRNAs for 72 h and AID-SPATA5 cells treated with 1 mM HU for 20 h were used as additional controls. n=3 biological replicates. **C.** Schematic and quantification of individual DNA fiber track lengths in HeLa S3 AID-SPATA5 cells treated with auxin for indicated times. Median is indicated. *p*-value determined by one-way ANOVA with Tukey’s multiple comparison test. N≥500 fibers sampled over three independent experiments. **D.** Schematic and quantification of replication fork restart assay in HeLa S3 cells treated with the indicated CRISPR guides for 8 days. Median is indicated. *p*-value determined by one-way ANOVA with Tukey’s multiple comparison test from n≥400 fibers sampled over three independent experiments. **E.** Quantification of proximity ligation assay (PLA) of PCNA and ADP-Ribose in HeLa S3 AID-SPATA5 cells treated with auxin for indicated times. PLA foci per nucleus are plotted and median is indicated. n≥400 cells sampled over 3 biological replicates. *p*-value determined by one-way ANOVA with Tukey’s multiple comparison test. **F.** Quantification of DAPI-stained HeLa S3 AID-SPATA5 cells treated with auxin for indicated times. HeLa S3 cells treated with indicated siRNAs for 72 h were used as additional controls. Data are presented as mean ± SEM, n≥3000 cells sampled over 3 biological replicates. *p*-value determined by unpaired Student’s *t*-test. **G.-H**. Representative images (**G**) and quantification (**H**) of cohesion defects upon auxin treatment in HeLa S3 AID-SPATA5 cells treated with indicated CRISPR guides for WAPL. Data are presented as mean ± SEM from 3 biological replicates and at least 50 metaphase spreads analyzed per sample per experiment. *p*-values derived from unpaired Student’s *t*-test for severe cohesion defects.

The 55LCC complex is a macromolecular machine that binds DNA, shows replication fork-stimulated ATPase activity, and associates with DNA replication factors on chromatin. To address whether 55LCC plays a specific role at replication forks, we performed iPOND (isolation of proteins on nascent DNA)^40^ and monitored 55LCC association in comparison to replisome components POLD3 and PCNA. SPATA5L1 was associated with actively replicating DNA in EdU pulldown experiments. However, it maintained EdU association after thymidine chase in contrast to POLD3 and PCNA (Figure 5A), indicating a more general 55LCC association with chromatin.

Given its essential nature (Figure 1H), we generated an auxin-inducible degron of SPATA5 to study the functions of 55LCC with enhanced temporal resolution. The auxin system recapitulated the loss of viability seen with CRISPR deletion of 55LCC (Figure S5A). However, 55LCC members were stable on chromatin, with residual protein being present even after 24 hours of auxin treatment (Figures 5B and S5B), which coincided with the onset of replication defects. Knockdown of 55LCC resulted in elevated replication stress indicated by an increase in phosphorylated RPA within 18–24 hours of auxin treatment (Figure 5B). In agreement, 55LCC loss decreased fork length upon 24 hours of auxin treatment with progressive shortening of DNA fiber lengths with increased auxin treatment times (Figure 5C). CRISPR deletion of SPATA5 and SPATA5L1 also led to replication fork stalling and decreased fork restart after hydroxyurea (HU) treatment and increased endogenous poly (ADP-ribose) (PAR) in S-phase cells upon 18–24 hours of auxin treatment (Figures 5D and E). Collectively, these findings indicate an increased presence of single stranded DNA during replication. Interestingly, the accumulation of replication-associated damage led to ATR checkpoint activation only after 72 hours of auxin treatment, commensurate with a G2 phase arrest (Figures 5B, S5C and D).

The absence of 55LCC produced a dramatic elevation in chromosome instability, with ~25% of cells showing micronuclei after 72 hours of auxin treatment (Figures 5F and S5E). Metaphase spreads upon auxin-induced degradation of SPATA5 revealed only a modest increase in DNA breaks (Figure S5F) but a severe loss of sister chromatid cohesion, characterized by the presence of railroad chromosomes (RR; chromatids lacking centromere constriction) and premature sister chromatid separation (PSCS)^41,42^ after 48–72 hours of auxin treatment (Figures 5G and H). Time course studies following auxin administration indicate that replication stress is the first observable phenotype upon loss of 55LCC and mitotic abnormalities, such as cohesion loss and micronucleation, arise at later time points of auxin treatment (~48–72h) (Figures 5B–H). This suggests that incomplete DNA replication could drive genome instability in subsequent cell cycles, consistent with recent reports on sister chromatid cohesion being dependent on DNA replication^43,44^. Under conditions of replication stress, cohesin removal by WAPL and PDS5B ensure fork progression and faithful DNA replication^45–47^. In accordance, we observed a moderately increased chromatin localization of WAPL upon loss of 55LCC (Figure S5G). Importantly, CRISPR-mediated knockdown of WAPL significantly reduced sister chromatid cohesion loss in 55LCC-deficient cells (Figure 5H), supporting the role of replication stress-associated cohesin remodeling in driving cohesion loss.

### 55LCC ATPase activity promotes cleavage of replisome factors in S-phase

55LCC suppresses DNA replication stress and mitotic chromosome instability. We posited these functions would derive from its putative unfoldase activity on interacting partner substrates. This hypothesis predicts that 55LCC substrates would become altered in a cell cycle-specific manner that required both 55LCC ATPase activity and the SPATA5 central pore loop aromatic residues, which are important for substrate engagement and unfolding in other unfoldases^37–39^.

**Figure 6:**
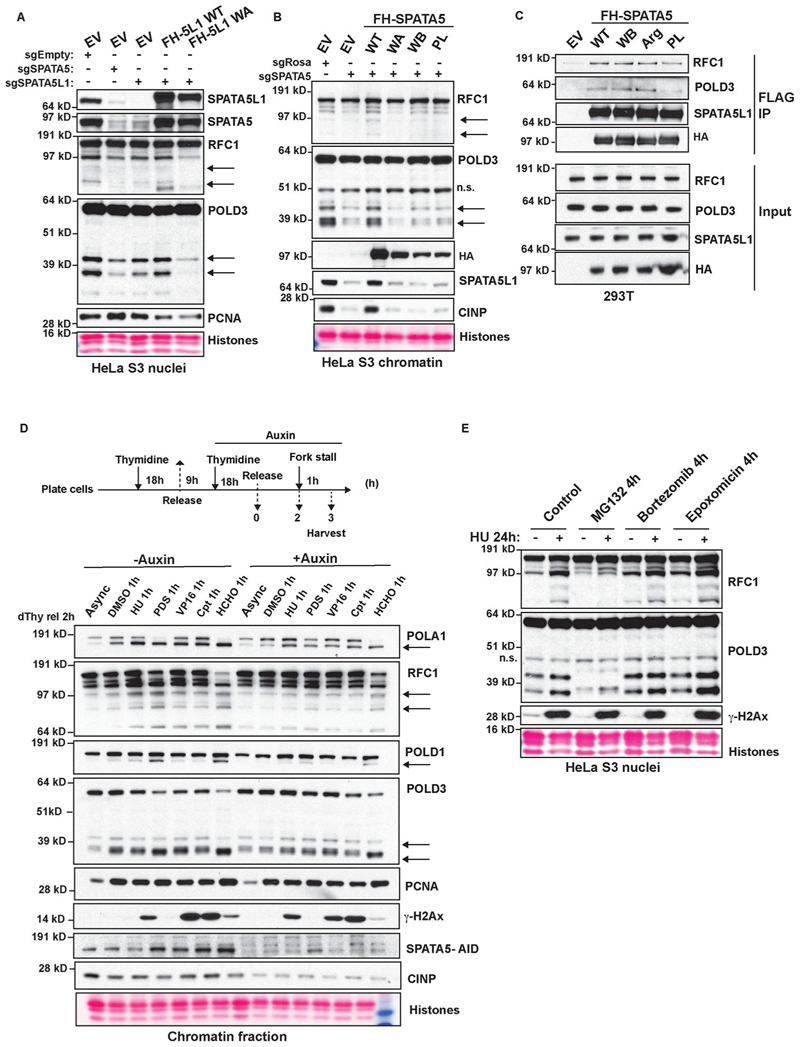
55LCC mediates cleavage of replication factors **A.** Western blot of acid extracted nuclei from HeLa S3 stably expressing FH-SPATA5L1 constructs or EV and treated with indicated CRISPR guides for 7 days. n=3 biological replicates. Arrows indicate cleaved products. Quantification and graphs in Table S5. **B.** Western blot of acid extracted chromatin from HeLa S3 cells stably expressing FH-SPATA5 constructs treated with the indicated guides for 8 days. WB = Walker B double mutant, Arg = Arginine finger double mutant, PL = Pore loop double mutant. n=3 biological replicates. n.s. indicates non-specific bands. Arrows indicate cleaved products. Quantification and graphs in Table S5. **C.** Western blot of FLAG IP and input samples from 293T cells transiently expressing FH-SPATA5 constructs or EV. n=3 biological replicates. **D.** Schematic and western blot of acid extracted chromatin from HeLa S3 AID-SPATA5 cells treated with −/+ Auxin for indicated time, released from double thymidine (dThy) for 2 h and treated with indicated drugs for 1 h. Async = Asynchronous. n=3 biological replicates. Arrows indicate cleaved products. Quantification and graphs in Table S5. **E.** Western blot of acid extracted nuclei from HeLa S3 treated with inhibitors for indicated times. n=3 biological replicates.

**Figure 7:**
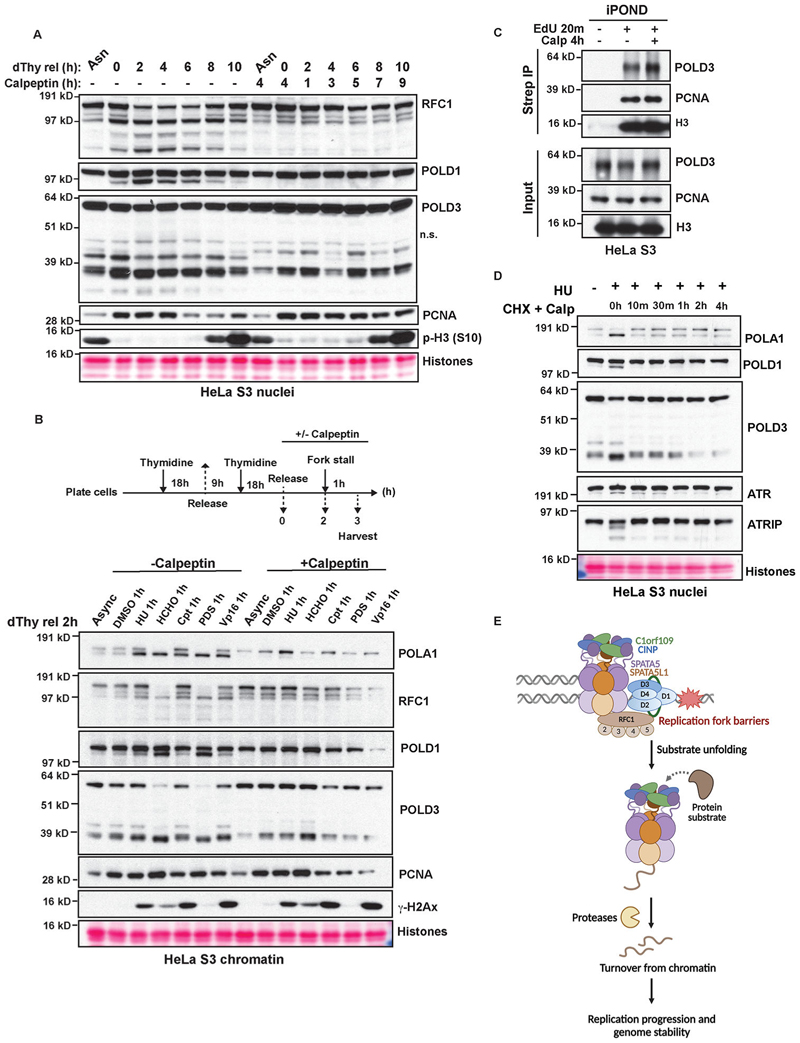
Cysteine protease-dependent proteolysis of the replisome **A.** Western blot of acid extracted nuclei from HeLa S3 released from double thymidine (dThy) and treated −/+ Calpeptin for indicated times. n.s. indicates non-specific bands. Asn = Asynchronous. n=3 biological replicates. Mean relative signal intensities of POLD3 and RFC1 full length proteins normalized to histones are quantified in Table S5. **B.** Schematic and western blot of acid extracted chromatin from HeLa S3 cells released from double thymidine (dThy) −/+ Calpeptin for 2 h and treated with indicated drugs for 1h. n=3 biological replicates. Async = Asynchronous. **C.** Western blot of Streptavidin IP and input of iPOND experiment from HeLa S3 cells treated with −/+ Calpeptin followed by −/+ EdU for indicated times. n=2 biological replicates. **D.** Western blot of acid extracted nuclei from HeLa S3 +/− 2 mM HU for 24 h followed by treatment with Calpeptin + Cycloheximide (CHX) for indicated times. n=3 biological replicates. **E.** Model depicting the function of 55LCC in unfolding and proteolysis of replisome components, thus maintaining genome stability.

To test these predictions, we performed western blots on chromatin fractions from HeLa S3 cells transduced with Rosa control or CRISPR guides against SPATA5 and SPATA5L1 (Figure 6A). 55LCC-interacting partners RFC1 and POLD3 displayed faster-migrating bands which we hypothesized to be cleavage products of the full-length proteins. These smaller peptides were diminished in the absence of 55LCC core constituents (Figures 6A and S5B, Table S5). Replisome cleavage required SPATA5 and SPATA5L1 Walker A and Walker B motifs, which are essential for nucleotide binding and ATPase activity (Figures 6A and B, Table S5). Additionally, cleavage products were diminished in the SPATA5 aromatic pore loop mutants, which also showed reduced substrate interaction by co-IP, consistent with central pore extrusion of substrates during unfolding (Figures 6B and C). Increased abundance of these lower molecular weight products occurred upon synchronization of cells in S and G2 phases with HU or RO-3306 treatment (Figures S6A and B). Double thymidine block confirmed cell cycle dependency of substrate processing (Figure 7A).

To determine conditions that increase 55LCC-dependent replisome cleavage, S-phase cells released from double thymidine block for 2 hours were subjected to different types of replication stress (Figures 6D and S6C). Increased processing and cleavage of replisome components, including POLD3, RFC1, POLA1, and POLD1 were observed on S-phase chromatin in response to different genotoxins and induced DNA lesions (Figures 6D and S6C, Table S5). These cleaved fragments were reduced within 18–20 hours of auxin-induced 55LCC degradation (Figure 6D and Table S5), suggesting 55LCC mediates replisome cleavage in response to replication fork barriers. In addition to replisome proteins, we observed processed fragments for ATR, ATRIP, and cohesin subunit RAD21 that were all reduced in the absence of 55LCC (Figures S5G and S6D). Replisome cleavage was unaffected by inhibition of transcription with alpha amanitin treatment (Figure S6E), blocking ribosome biogenesis with chemical perturbation of RNA pol I transcription with BMH-21 and CX-5461^48,49^ (Figures S6F and G), or knockdown of ribosome recycling factor RSL24D1, which is a 55LCC substrate^50^ (Figure S6H). RSL24D1 loss also did not cause increased micronucleation or G2/M arrest (Figures 5F and S5D), suggesting that the effect of 55LCC on DNA replication and genome stability is likely independent of its reported ribosomal function. Replisome cleavage was decreased to varying extents in the EHLMRS patient mutants (Figures S6I and J). However, SPATA5 and SPATA5L1 patient mutations, with the exception of SPATA5L1 I466M and G689V, exhibited reduced stability and were unable to maintain a stable 55LCC complex upon CRISPR-knockout of the endogenous wild-type counterpart (Figures S6I and J).

### Replisome cleavage is ubiquitin- and proteasome-independent

The appearance of faster-migrating products for multiple replisome substrates was investigated in HU-treated cells in combination with either p97 inhibitor NMS873 or proteasome inhibitor MG132, or with ATR kinase inhibitor VE821. No reductions were observed with ATR and p97 inhibition (Figures S7A), consistent with a distinct proteostasis mechanism driven by 55LCC activity in response to replication stress (Figure S7A). In contrast, treatment with the broad protease inhibitor MG132 resulted in evident reductions, confirming these fragments are indeed the result of proteolytic cleavage (Figures 6E, S7A, and B). We extended investigation to the more selective proteasome inhibitors, bortezomib and epoxomicin, which did not reduce levels of the cleaved products compared to controls (Figures 6E and S7C). Replisome cleavage was unaffected upon inhibition of E1 ubiquitin-activating enzymes (Figure S7C), providing further evidence against a ubiquitin-proteasome-mediated event, and suggestive of an off-target activity for MG132 in reducing substrate cleavage.

### Cysteine protease-dependent cleavage of 55LCC substrates

MG132 is known to also inhibit the calpain family of Ca^2+^-activated cysteine proteases^51^. Calpain inhibitors calpeptin and ALLN prevented replisome cleavage in S-phase and in response to replication stress (Figures 7A, B, and S7D–F). Moreover, we reproducibly observed increased replisome cleavage with agents that increase Ca^2+^ flux, digoxin and ouabain, which was reversed by calpeptin treatment or CRISPR deletion of SPATA5 or SPATA5L1 (Figures S7F and G). CRISPR knockout of Calpain 1, or Calpain 1/2 common small subunit CAPNS1, reduced RAD21 cleavage^52^ and also partially affected cleavage of POLD3 (Figure S7H). This suggests that 55LCC-mediated unfolding creates accessibility of the unfolded substrates to Calpain 1, and possibly other cysteine proteases. Importantly, calpeptin treatment increased chromatin retention of full-length replisome proteins, POLD3, RFC1, and PCNA from S-phase through G2/M phases of the cell cycle (Figure 7A and Table S5) and on replicating chromatin as detected by iPOND (Figure 7C).

Non-processive proteolysis by cellular proteases generates peptides that are often short-lived and targeted for proteasomal clearance by N- and C-degron pathways^53–56^. We treated cells with HU to increase the abundance of the cleaved products and examined the fate of these replisome cleaved products on chromatin using a cycloheximide pulse assay in the presence of calpeptin to prevent *de novo* cleavage events. Most of the cleaved products of POLD3, POLD1, POLA1, ATR kinase, and ATRIP disappeared from chromatin within 10 minutes of cycloheximide addition (Figure 7D). Cleaved products of RAD21 were also rapidly removed from chromatin upon cycloheximide and calpeptin addition (Figure S7I). These data are indicative of 55LCC and cysteine protease-mediated cleavage being involved in the clearance of replisome proteins from chromatin.

## Discussion

We identify the macromolecular AAA+ ATPase complex, 55LCC, that processes replisome substrates and regulates genome stability. SPATA5 and SPATA5L1 assemble into a DNA-binding stacked ring heterohexamer that constitutively interacts with the C1orf109-CINP heterodimer via the SPATA5 and SPATA5L1 N-termini. This four-membered complex associates with specific replisome proteins and mediates their ATPase-dependent processing in response to replication stress. We propose that 55LCC acts as an unfoldase, thereby remodeling protein complexes and facilitating their subsequent cleavage by cysteine proteases and removal from chromatin. Ubiquitin-independent 55LCC unfoldase-coupled proteolysis and turnover from chromatin represents an important mechanism of replication factor regulation that is critical for replication fork progression and genome integrity (Figure 7E).

SPATA5 and SPATA5L1, along with p97, belong to the classic clade of AAA+ ATPases involved in protein remodeling and degradation^20,57,58^. Interactions between p97 and its cofactors are largely mediated via the N-terminal domain of p97^26–28,59^. Similarly, the SPATA5 and SPATA5L1 N-termini were required for binding to C1orf109-CINP and for association into a stable heterohexameric complex (Figures 3A and S4G–I). However, while p97 largely binds ubiquitylated substrates^60–64^, 55LCC likely recognizes substrate proteins in a ubiquitin-independent manner. Furthermore, 55LCC ATPase activity is specifically enhanced by DNA structures while p97-mediated ATP hydrolysis is unaffected by nucleic acids (Figures 2E–H). These observations differentiate 55LCC from p97 and suggest that the regulatory mechanisms for their enzymatic functions are likely different.

The 55LCC complex structure contains two copies of C1orf109-CINP that together with the SPATA5 and SPATA5L1 NTDs form a lid unit on the top of the SPATA5-SPATA5L1 ATPase motor (Figure 3A). As such, 55LCC is the only heterohexameric ATPase motor to date with a 4:2 stoichiometry. Conservation analysis of aromatic pore loop residues (required for substrate engagement and threading)^27,29,39^ suggests differences between SPATA5 and SPATA5L1 (Figure S2B). SPATA5 has conserved aromatic pore loop residues in both ATPase 1 and 2 domains whereas only ATPase 2 pore loop residues are conserved in SPATA5L1 (Figure S2B). Variability in these residues has been linked to different substrate processing mechanisms of other AAA+ ATPases^65–68^ and the combination and precise stoichiometry in 55LCC suggest a synergistic role between SPATA5 and SPATA5L1 activities. Consistent with this idea, the full 55LCC complex has higher ATPase activity than the SPATA5 homohexamer (Figures 2E–H, *top*).

55LCC members were present on replicating chromatin (Figure 5A), and 55LCC-dependent substrate cleavage was highest in S phase and enhanced by replication fork damage (Figures 6D and S6A–C, Table S5). Additionally, 55LCC ATPase activity was only stimulated by replication fork DNA (Figures 2E–H, *top*). Replication stress and fork progression defects were the earliest observable phenotypes upon 55LCC loss, while cohesion loss and micronucleation arose in subsequent cell cycles (Figures 5B–H). We posit that this chromosomal instability emanates from replication defects, consistent with recent studies that indicate the importance of completing DNA replication in establishing sister chromatid cohesion^43,44^.

We identify extensive processing of replisome components on chromatin, including DNA polymerase δ, α, and clamp loader RFC complexes (Figures 6A, B, D, and S5B). These substrates interact with 55LCC and require its putative unfoldase functionalities for processing (Figures 1C, S1A, B, and 6A–C, Table S5). We propose that altered proteostasis of replisome components at replication fork impediments contributes to the increased replication stress and subsequent genome instability in 55LCC-deficient cells. Unfolded replisome substrates could be targeted for cleavage by an array of chromatin-localized proteases including cysteine proteases, such as calpains, and histone proteases, such as cathepsins^52,69^.

Germline mutations in SPATA5 and SPATA5L1 were identified in a spectrum of neurodevelopmental disorders characterized by microcephaly, intellectual disability, seizures, spasticity, hearing loss, and thrombocytopenia^13–18^. Similar phenotypes are observed in diverse human syndromes that arise from loss of function mutations in genes that ensure DNA replication fidelity^70^. Structural analyses coupled with cell-based reconstitution of SPATA5 and SPATA5L1 patient mutants demonstrates that these disease variants affect 55LCC stability, assembly, and enzymatic processing of the replisome (Figures 4, S6I and J, Table S4, Movie S1). Given the position of C1orf109-CINP within the complex, we hypothesize that additional, and yet to be discovered, disease mutations will also affect C1orf109-CINP interactions with SPATA5-SPATA5L1 and contribute to complex destabilization. Alterations in 55LCC-dependent replisome processing and the resultant genome instability may contribute to a subset of phenotypes in these disorders. Supporting this idea, a recent study using EHLMRS patient-derived mutant fibroblasts implicated SPATA5L1 in DNA replication and mitosis^18^.

Our data suggest an expansive cellular role for 55LCC unfoldase activity outside of DNA replication and repair, as this complex interacts with key components of the ribosome and proteasome, among others (Figure 1D). The SPATA5 ortholog in yeast, Drg1, is involved in ribosome biogenesis by recycling the shuttling factor Rlp24^71–73^. A divergent N-terminus and the absence of the other 55LCC members in yeast suggest that this complex must have evolved diverse functionalities in higher organisms, although its role in ribosome biogenesis is conserved as reported recently^50^. Our data indicates that replisome functions of 55LCC are likely independent of its functions in ribosome biogenesis (Figures 5F, S5D, and S6F–H). Collectively, this raises the possibility that 55LCC, and perhaps other proteostasis control mechanisms, co-regulate DNA replication and protein synthesis in proliferating cells^74–76^.

### Limitations of the study

We propose that 55LCC is an active unfoldase based on requirements for both ATPase activity and aromatic pore loop residues in replisome cleavage. Being unable to confirm the unfoldase activity *in vitro*, however, is a limitation of the study. Demonstrating unfoldase activity of p97 involved ~30 years of structural, biochemical, and cell biology studies characterizing principles of substrate recognition by p97^26,37,59,62,77,78^. The signals responsible for substrate unfolding by 55LCC and its regulatory mechanisms remain undefined and are important avenues for future investigation. Our ability to fully assemble the 55LCC complex will facilitate the development of systems to reconstitute unfoldase activity and determine how replication fork DNA and/or other factors enhance 55LCC activity. Our structural studies have revealed how SPATA5-SPATA5L1 and C1orf109-CINP assemble into a heterohexameric 55LCC ATPase complex. However, the resolution of the reported model (4.0–4.5 Å) and its inherent flexibility currently limits mechanistic insights to residues that have a structural integrity function within a folded domain, leaving disease mutations on more flexible areas harder to rationalize. We identify replication factor proteolysis mediated by putative 55LCC unfoldase activity upon replication stress. We have included quantifications of the chemiluminescent immunoblot data across replicates (Table S5) that consistently demonstrates 55LCC activity in processing replisome substrates during replication stress. We note that treatment with different genotoxins affects cleavage of replisome components to varying levels (Figure 6D), suggesting the involvement of additional pathways, proteases, or covalent modifications that warrant further investigation.

## STAR Methods

### Resource availability

#### Lead Contact

Further information and requests for resources and reagents should be directed to and will be fulfilled by the lead contact, Roger Greenberg (rogergr@pennmedicine.upenn.edu).

#### Materials availability

All newly generated reagents and cell lines generated in this study are available upon request from the lead contact.

### Experimental model and study participant details

#### Mammalian cell culture

U-2 OS, HeLa S3, HEK293T, and Phoenix cell lines were grown in DMEM (Thermo Fisher Scientific) with 10% bovine calf serum (Fisher Scientific /GE Hyclone) and 1x penicillin/streptomycin (Gibco). MCF 10A cell line was grown in a 1:1 mixture of F12:DMEM media with 5% horse serum (Thermo Fisher Scientific), 20 ng/ml human Epifermal Growth Factor (Peprotech), 0.5 μg/ml hydrocortisone (Sigma), 100 ng/ml cholera toxin (Sigma), and 10 μg/ml recombinant human insulin (Sigma). HCT116 cell line was grown in McCoy’s 5A media (Thermo Fisher Scientific) supplemented with 10% bovine calf serum (Fisher Scientific /GE Hyclone) and 1x penicillin/streptomycin (Gibco). Cell lines were maintained at 37°C with 5% CO2. All cell lines were routinely tested for mycoplasma contamination using MycoAlert Mycoplasma Detection kit (Lonza, LT07–518). Following antibiotics were used for stable cell generation -puromycin (2 μg/ml), blasticidin (10 μg/ml), and G418 (400 μg/ml).

#### Bacterial and insect cell cultures

Bacterial cells were grown in Luria-Bertani (LB) broth (Fisher Chemical) or Terrific Broth (TB; Fisher Chemical) media supplemented with appropriate antibiotics [100 μg/ml ampicillin (Merck Life Science), 34 μg/ml chloramphenicol (Amresco), 10 μg/ml gentamycin (VWR), 50 μg/ml kanamycin (Thermo Fisher Scientific), 10 μg/ml tetracycline (Sigma), 50 μg/ml spectinomycin (Merck Life Science)], and incubated at 37°C and 18°C as per requirements. For preparation of bacmids DNA, x-gal (Promega UK) and isopropyl-β-D-1-thiogalactopyranoside (IPTG; Fluorochem) were also added to bacteria cultures at final concentrations of 15 μg/ml and 40 μg/ml, respectively. *Spodoptera frugiperda 9* (*Sf9*) insect cells (Thermo Fisher Scientific) were grown in SF 900 II SFM media (Thermo Fisher Scientific) supplemented with 1x antibiotic-antimycotic mix (Thermo Fisher Scientific), and maintained at 27°C.

### Method details

#### Plasmids and CRISPR-Cas9 sgRNA cloning

Expression vectors were generated by cloning cDNA (Biosettia, cDNA-hsa-03) (POLD3 RefSeq NM_006591.2, SPATA5L1 RefSeq NM_024063.3, SPATA5 RefSeq NM_145207.3, C1orf109 Refseq NM_017850.3, CINP RefSeq NM_032630.3) into the pOZ-N-FLAG-HA retroviral vector using standard protocols. Mutants were generated using Q5 site-directed mutagenesis kit (NEB, E0554S). CRISPR lines were generated using a two-vector system (pLentiCas9 and pLentiGuide) and selected for indicated times. sgRNAs were designed to target the start codons or the functional domains of the genes^79^. Complementary oligos were annealed and ligated into BsmBI-digested pLentiGuide vector. Auxin-inducible degron (AID)-tagged SPATA5 was generated by cloning EGFP-AID tag into pOZ-N-FLAG-HA retroviral vector followed by insertion of CRISPR resistant SPATA5 cDNA (crR SPATA5) to the C-terminus of AID tag using standard protocols. A list of all primers and sgRNAs used in the study is provided in Table S6.

Transient plasmid and siRNA transfections were carried out with LipoD293 (Signagen, SL100668) or Lipofectamine 2000 (Invitrogen, 11668019), respectively, according to manufacturer’s instructions. Analyses were performed 24–48 hours (h) after transfection of plasmids, and 72 h after siRNA transfection. All siRNAs were used at a final concentration of 25 nM.

#### Lentivirus generation and transduction

Lentivirus was generated by co-transfection of HEK293T cells with the lentiviral plasmid of interest and packaging plasmids pMD2.g (VSVG), pRSV-Rev, PMDLG/pRPE in the ratio of 2:1:1:1 using LipoD293 (Signagen). Virus was collected 48 and 72 h post-transfection and syringe-filtered (0.45 μm) and used to transduce target cells with 8 μg/ml polybrene (Sigma, H9268). Transduced cells were selected with antibiotics.

#### Retrovirus generation

For generating retrovirus, Phoenix cells were transfected with the target retroviral vector and pCL ampho in a ratio of 2:1 using LipoD293. Virus was collected at 48 and 72 h post-transfection and syringe-filtered and used to spin-infect target cells with 8 μg/ml polybrene at 500 g for 30 minutes (min). pOZ constructs were selected using interleukin-2 (IL-2)-conjugated magnetic beads.

#### CRISPR Cas9 knockout generation

All knockouts were generated using CRISPR-Cas9. Lentiviral transductions were performed, followed by antibiotic selection to generate a stable knockout cell line. Independent lentiviral infection and antibiotic selection was performed for all biological replicates.

#### Generation of auxin-inducible SPATA5 cell line

HeLa S3 cells stably expressing pBABE TIR1–9myc Puro and pOZ-N-EGFP-AID-crR SPATA5 were generated by retroviral transduction followed by selection with puromycin and (IL-2)-conjugated magnetic beads, respectively. Endogenous SPATA5 was knocked out using CRISPR Cas9 with a single vector system (pLentiCRISPRCas9v2) and single clones were selected. Expression of TIR-1–9 myc and EGFP-AID-SPATA5 and endogenous SPATA5 knockout were confirmed by western blotting.

#### Cell fractionation and Western blot

For whole cell lysates, cells were lysed in RIPA buffer supplemented with MgCl_2_, 50 U/ml benzonase (Millipore, 70746–3), and complete EDTA-free protein inhibitor cocktail (Roche). Cell fractionations were performed as previously described^80^. Cytoplasmic fractions were obtained by lysing cells in a buffer containing 10 mM HEPES pH 7.4, 10 mM KCl, 0.05% NP-40. Nuclear pellets were then processed in two different ways. Whole nuclear extracts were obtained by treating nuclei with 0.2 N HCl and subsequent neutralization with 1 M Tris pH 8.0. Alternatively, nuclear fractionation was carried out with low salt buffer containing 10 mM Tris-HCl pH 7.4, 0.2 mM MgCl_2_, 1% Triton-X-100 to obtain nuclear soluble fraction followed by treating the pellet with 0.2 N HCl to obtain chromatin fraction. All fractionation steps were performed on ice for 20 min with 0.5 mM PMSF. After each step, samples were spun at maximum speed at 4°C for 10 min. Western blotting was performed using standard conditions, as previously described^11^. Ponceau staining was used to visualize histones and confirm chromatin extraction. Chemiluminiscent western blotting data from chromatin fraction were quantified using Fiji^81^ and normalized to histones.

#### Immunoprecipitation

For whole cell co-immunoprecipitation, cells were lysed in IP buffer (100 mM NaCl, 50 mM Tris-HCl pH 7.4, 0.2% NP-40, 10% glycerol, 1 mM MgCl_2_, 1 mM DTT) supplemented with complete EDTA-free protease inhibitor cocktail (Roche) and 50 U/ml Benzonase (Sigma, 70746–3) and incubated at 4°C for 1 h under end-over-end rotation. After nuclease digestion, lysates were cleared by centrifugation. FLAG IP was performed using anti-FLAG M2-agarose beads (Sigma, A2220) for 2 h at 4°C followed by FLAG peptide (Sigma, F4799) elution or elution with 0.1 M glycine pH 2.5 and neutralization.

#### FLAG-HA purification and mass spectrometry

FLAG-HA tandem purification from nuclear extracts was performed as previously described^82^. Briefly, nuclei were isolated using hypotonic buffer (10 mM Tris-HCl pH 7.4, 7.5 mM KCl, 1.5 mM MgCl_2_) with douncing. Nuclei were subsequently lysed in KETNG400 buffer (400 mM KCl, 20 mM Tris-HCl pH 7.4, 0.5 mM EDTA, 0.1% NP-40, 1.5 mM MgCl_2_, 10% glycerol, 0.5 mM PMSF, and 5 mM β-mercaptoethanol) and dialyzed overnight at 4°C in KETNG150. FLAG IP was performed using anti-FLAG M2-agarose beads (Sigma, A2220) for 2 h at 4°C followed by FLAG peptide (Sigma, F4799) elution. HA IP was performed using EZView Red HA beads (Sigma, E6779) for 2 h at 4°C followed by elution with 100 mM glycine, pH 2.5 and neutralization. 10–20% of the final eluate was electrophoresed on a 4–12% bis-tris SDS gel (Invitrogen) and silver-stained (Invitrogen, LC6070). The remaining eluate was precipitated with trichloroacetic acid (TCA) using standard conditions. The lyophilized eluate was submitted to the Taplin Mass Spectrometry Facility at Harvard University for mass spectrometry analysis.

#### Immunofluorescence and microscopy

Immunofluorescence was performed using standard conditions, as previously described^11^. Micronuclei were visualized using 4′,6-diamidino-2-phenylindole (DAPI) stain and images were analyzed using Fiji^81^. Total number of cells in the image was quantified using Cell Profiler 4.2.5^83^ (Cell Profiler Module: ColorToGray/IdentifyPrimaryObjects).

#### Proximity ligation assay (PLA)

This protocol was adapted from previously described protocol^84^. To detect S-phase PARylation, cells were seeded onto coverslips and treated with auxin for indicated times. Before harvesting, cells were treated with 10 μM PARG inhibitor (PDD00017273, Tocris) for 30 min. All the downstream harvesting up to primary antibody staining was done in pre-chilled reagents to which 10 μM PARG inhibitor and 10 μM olaparib were added. Coverslips were rinsed with PBS and pre-extracted with ice-cold cytoskeletal (CSK) buffer (10 mM HEPES pH 7.4, 300 mM sucrose, 100 mM NaCl, 3 mM MgCl_2_) containing 0.5% Triton-X-100 for 5 min followed by quick washes with PBS and fixation with ice-cold 4% paraformaldehyde for 15 min. Cells were rinsed with PBS and permeabilized in 1:1 Methanol/Acetone mix on ice for 5 min followed by 0.5% Triton-X-100 in PBS for 5 min on ice. Coverslips were then washed twice with PBS, blocked in 3% BSA in PBS for 1h followed by incubation with PAR (1:200 in 3% BSA in PBS) and PCNA antibodies (1:500 in 3% BSA in PBS) overnight at 4°C. No primary antibody added was used as a negative control. After overnight incubation, coverslips were washed in Duolink wash buffer A (Sigma-Aldrich, DUO82049) three times for 5 min each. PLA PLUS and MINUS probes (Sigma-Aldrich, DUO92004 and DUO92002) were diluted 1:5 in blocking buffer and applied to the coverslips followed by incubation in a pre-heated humidity chamber for 1h at 37°C. PLA signals were detected using Duolink In Situ Detection Reagents (Sigma-Aldrich, DUO92008). Coverslips were washed with wash buffer A three times for 5 min each followed by addition of Duolink ligation buffer containing the ligase at 1:40 dilution and incubation at 37°C for 30 min in the pre-heated humidity chamber. Polymerase diluted in Duolink Amplification bufffer at 1:80 was applied to the coverslips after 2 washes with wash buffer A and incubated at 37°C for 100 mins in the pre-heated humidity chamber. Coverslips were then washed thrice in wash Buffer B for 10 min each at room temperature (RT), stained with DAPI (1 μg/ml in PBS) for 15 min, washed in PBS and mounted with Prolong Gold (ThermoFisher, P36934). Images were acquired on CoolSnap Myo camera attached to Nikon eclipse 80i microscope and NIS-elements software. Images were analyzed using CellProfiler 4.2.5 to identify and measure PLA and DAPI signals (Cell Profiler Modules: IdentifyPrimaryObjects/MeasureObjectIntensity) PLA signal was masked with DAPI signal to ensure only nuclear signals were quantified (Cell Profiler Modules: MaskObjects). To identify PLA signals from each nucleus, RelateObjects module was used and number of PLA foci from each nucleus was obtained and quantified using Prism.

#### Clonogenic survival assay

For clonogenic assays, CRISPR guide viruses were added to cells and selected for 4–7 days prior to seeding. Cells were seeded at 300 and 500 cells per well in 6-well plates (technical triplicates) and allowed to grow for 7–14 days. For experiments with AID-SPATA5 lines, cells were plated with and without auxin for 7–10 days. Plates were then stained with crystal violet for 30 min, washed with deionized water, and dried. Colonies were counted using Fiji^81^.

#### DNA fiber spreading

For quantifying DNA fiber track lengths, cells seeded in 6-well plates were incubated with 20 μM CldU (Sigma, C6891) for 15 min at 37°C followed by one quick wash with PBS and incubation with 200 μM IdU for 15 min at 37°C. Cells were then washed with cold PBS at least twice, trypsinized, counted, centrifuged at 3000 rpm for 3 min and resuspended to a final concentration of 10^6^ cells/ml. 2 μl of this cell suspension was added onto the edge of silane-coated slides (Newcomer Supply, 5070) and allowed to rest for 1 min followed by addition of 10 μl lysis buffer (200 mM Tris-HCl pH 7.4, 50 mM EDTA, and 0.5% SDS). After 2 min the slides were tilted at an angle of 20–30° and the DNA was allowed to spread on the slides under gravity. Slides were air-dried, fixed in methanol:acetic acid (3:1) solution for 10 min, air-dried and stored at −20°C. Slides were then washed once in PBS, denatured in 2.5M HCl for 1h at RT and neutralized in 400 mM Tris-HCl pH 7.4 for 10 min. Slides were then washed in PBS and PBST for 3–5 min and blocked in 5% BSA + 10% goat serum for 1 h. Primary antibodies (anti-IdU: 1:20 dilution, 347580, BD Pharmigen; anti-CldU: 1:100 dilution, ab6326, Abcam) were applied to the coverslips for 1h at RT in a humidity chamber followed by washes and incubation with secondary antibodies for 1 h. Slides were then washed thrice in PBST and mounted with Prolong Gold (ThermoFisher, P36934). For fork restart assay, cells were treated with 50 μM IdU for 20 min, followed by 2 mM HU (Sigma) for 1 h. After washes, cells were incubated with 250 μM CldU for 20 min prior to harvesting and staining as described above. Images were captured using 63X objective on CoolSnap Myo camera attached to Nikon eclipse 80i microscope and NIS-elements software. Images were analyzed using Fiji^81^.

#### Metaphase Spread

HeLa S3 AID-SPATA5 cells were treated in the presence or absence of auxin for the indicated time. Prior to harvesting, 0.1 μg/ml of colcemid was added to cells for 2 h. Cells were harvested by trypsinization, washed with PBS, and pellets were swelled in pre-warmed 0.075 M KCl at 37°C for 15–20 min. Cells were then fixed with freshly prepared methanol to acetic acid (3:1 v/v) solution at 4°C overnight. Metaphase spreads were performed by dropping the fixed cells onto glass slides rinsed with 50% acetic acid.

Slides were air-dried and stained with Giemsa staining buffer for 3–5 min at RT, washed, and mounted with Permount. Images were acquired using Olympus BX-41 microscope connected to DP25–4 camera. Images were analyzed using Fiji^81^.

#### Cell cycle analysis and flow cytometry

Cells were trypsinized and washed with PBS followed by fixation in 70% ethanol at −20°C overnight. Cells were then centrifuged at 500 g, 5 min, 4°C and resuspended in 500 μl PBS containing RNase A (100 μg/ml) and propidium iodide (50 μg/ml). Cells were stained at RT for 30 min. The samples were analyzed on a MACSQuant VYB flow cytometer. Data were analyzed using FlowJo software.

For phospho-Histone H3 (Ser 10) staining, cells were trypsinized, washed with PBS and fixed in 4% paraformaldehyde in PBS for 15 min at RT followed by washes with 1% BSA in PBS. Cells were then permeabilized with 0.5% Triton-X-100 in PBS for 5 min at RT followed by washes with 1% BSA in PBS. Cells were then stained with phospho-Histone H3 (Ser10) antibody for 1h and 37°C followed by washes with 1% BSA in PBS containing 0.1% NP-40 and secondary antibody staining for 1h and 37°C. Cells were then washed and resuspended in 1% BSA in PBS + 0.1% NP-40, 1 μg/ml DAPI and 100 μg/ml RNase overnight at 4°C^85^. The samples were analyzed on a MACSQuant VYB flow cytometer. Data were analyzed using FlowJo software.

#### iPOND

iPOND was performed as previously described^86^. Briefly, ~100 X 10^6^ asynchronously growing HeLa S3 cells were pulse-labeled with 10 μM EdU (Thermo FIsher Scientific, E10187) for 20 min followed by fixation using 1% formaldehyde for 20 min at RT. Formaldehyde fixation was quenched by 0.125 M glycine and cells were collected in 50-ml conical tubes by scraping with a cell lifter. Cells were pelleted by centrifugation at 900 g for 5 min at 4°C and washed three times with 1X ice-cold PBS. Cell pellets were resuspended in permeabilization buffer (0.25% Triton X-100 in PBS) and incubated at RT for 30 min. Cells were pelleted and washed once with cold 0.5% BSA in PBS by centrifugation at 900 g for 5 min at 4°C. Cell pellets were resuspended in 1X Click reaction cocktail (10 μM Biotin-azide (B10184, Thermo Fisher Scientific), 10 mM Sodium ascorbate, 2 mM CuSO4 in 1X PBS) and incubated at RT for 2 h with rotation. Cells were pelleted and washed once with 1X PBS by centrifugation at 900 g for 5 min at 4°C followed by resuspension in lysis buffer (1% SDS in 50 mM Tris, pH 8.0). Chromatin was solubilized using a microtip probe sonicator with 5 pulses at 30% amplitude with 20 S on/40 S off setting. Samples were centrifuged at 16,100 g for 10 min at RT, supernatant was collected and filtered through a 100-micron nylon mesh. Filtered samples were incubated with 50 μl streptavidin agarose beads (EMD Millipore, 69203) for 2 h at RT with rotation. Beads were collected by centrifugation at 1800 g for 3 min at RT followed by extensive wash with lysis buffer and 1 M NaCl. iPOND captured proteins were eluted by boiling the beads in 2X SDS sample buffer at 95°C for 30 min and analyzed by SDS-PAGE followed by western blotting.

#### RNA isolation and qPCR

RNA was isolated using RNAeasy mini kit (Qiagen, 74104) according to manufacturer’s protocol. cDNA synthesis was carried out with High-Capacity cDNA Reverse Transcription Kit (Thermo Fisher Scientific, 4368814) using 2 μg of total RNA, according to manufacturer’s instructions. 10 μl qPCR reactions were set up in triplicates with Power SYBR Green master mix (Thermo Fisher Scientific, 4367659) using 20 ng cDNA per reaction. The reactions were run on Applied Biosystems QuantStudio 6 Pro system and Design and Analysis software 2.6.0 was used for analysis using relative quantification and standard module. 18s rRNA was used as internal reference control. Primers used are in Table S6 ^87,88^.

#### Phylogenetic analysis

Amino acid sequences of SPATA5 (Q8NB90) and SPATA5L1 (Q9BVQ7) were queried using BlastP^89^ against human proteins in the nonredundant (nr) database^90,91^. Multiple sequence alignment of the identified and selected proteins was performed using MUSCLE software^92–94^. Maximum likelihood phylogenetic trees were generated using MEGA software version 10.1.7 with LG amino acid substitution matrix and Gamma distribution model and 100 bootstrap replicates^95,96^. Trees were visualized and annotated using iTOL (https://itol.embl.de/).

### Generation of plasmid constructs

#### Cloning and expression of proteins and complexes in insect cells

To generate constructs for insect cells expression, genes for human full-length (FL) SPATA5 wild-type (WT), FL SPATA5L1 WT and FL C1orf109 WT were cloned into pUCDM, pFL and pSPL vectors respectively, and assembled into a single multigene cassette via Cre-mediated recombination^97^. A 6xHis, Flag and double (d)StrepII-ybbR^98^ purification tags, each followed by a Tobacco Etch Virus (TEV) protease site, were engineered at the N-termini of SPATA5, SPATA5L1, and C1orf109 respectively. Site-directed mutagenesis was used to generate the Walker B double mutants (WB) of SPATA5 and SPATA5L1, using FL SPATA5 WT in pUCDM and FL SPATA5L1 WT in pFL as templates. All mutations were confirmed by DNA sequencing, and the resulting SPATA5 and SPATA5L1 variants were assembled into a single multigene construct as described above. The DNA sequences for human FL SPATA5 WT, FL C1orf109 WT and FL CINP WT were cloned into pFastBac-HTB using standard molecular biology techniques. Both SPATA5 and CINP constructs bear a 6xHis purification tag and TEV protease site at the N-terminus, while C1orf109 is also characterized by a dStrepII-ybbR tags in between the 6xHis and TEV site. A list of primers used in this study is summarized in Table S6.

Bacmid DNA was generated in DH10 MultiBac^*Turbo*^ cells (ATG Biosynthetics) following manufacturer’s protocol, and virus amplification in *Sf9* cells (Thermo Fisher Scientific) was performed using standard procedures and as previously described^99^. For recombinant expression of SPATA5 and C1orf109, *Sf9* cells were infected with baculoviruses encoding each construct. To express the SPATA5-SPATA5L1-C1orf109-CINP complexes (with WT or WB forms of SPATA5-SPATA5L1; here referred to as 55LCC WT or WB, respectively), *Sf9* cells were co-infected with baculoviruses encoding SPATA5-SPATA5L1-C1orf109 variants and CINP in a 1:1 ratio. Following 48 h after infection, cells were harvested by centrifugation at 500 g for 15 min and protein purification was carried out as outlined below.

#### Cloning and expression of proteins in bacteria

For protein expression in bacteria, the genes encoding human FL CINP and SPATA5 1–336 were cloned into pProEx-HTB using standard techniques. Site-directed mutagenesis was used to generate the disease variants of SPATA5 1–336, using the WT construct as template (see Table S6 for a list of primers used). All mutations were confirmed by DNA sequencing. An expression construct for human FL VCP/p97 cloned into pET15b was a gift from Hemmo Meyer (Addgene plasmid #169020)^60^.

WT and mutant proteins were expressed with an N-terminal 6xHis tag in *Escherichia coli* (*E. coli*) BL21 (DE3) RIL cells (Thermo Fisher Scientific). Cells were grown at 37°C in TB medium to an OD_600_ of ~1.6, then induced with 0.5 mM IPTG and grown overnight (O/N) at 18°C. Cells were harvested by centrifugation at 6,000 g for 20 min, and protein purification was carried out as described below.

### Protein purification

#### Purification of 55LCC variants

Cells expressing 55LCC were resuspended in 100 ml ice-cold lysis buffer [50 mM Tris-HCl pH 7.6, 300 mM NaCl, 20 mM Imidazole, 5% (v/v) glycerol, 1 mM ethylene glycol-bis(β-aminoethyl ether)-N,N,N’,N’-tetraacetic acid (EGTA), 2 mM adenosine diphosphate (ADP), 2 mM MgCl_2_, 0.075% (v/v) β-mercaptoethanol and 1 mM benzamidine] supplemented with two tablets of Pierce^™^ protease inhibitor (Thermo Fisher Scientific) and lysed by sonication using a Sonics Vibracell instrument (1 s ON/3 s OFF, at 40% amplitude for 4 min). The cell lysate was cleared by centrifugation at 30,000 g for 30 min at 4°C, and the soluble fraction was sonicated (1 s ON/3 s OFF, at 40% amplitude for 2 min) and subsequently passed through a 0.45 μm filter (Fisher Scientific). The sample was then incubated with 3 ml HisPur^™^ Ni-NTA resin (Thermo Fisher Scientific), pre-equilibrated in low salt buffer [50 mM Tris-HCl pH 7.6, 300 mM NaCl, 20 mM Imidazole, 5% (v/v) glycerol, 1 mM EGTA, 2 mM ADP, 2 mM MgCl_2_, 0.075% (v/v) β-mercaptoethanol and 1 mM benzamidine], for 1 h at 4°C in rotation. After washing the resin with 20 ml low salt buffer, 20 ml high salt buffer (low salt buffer containing 500 mM NaCl), and 20 ml low salt buffer, the complex was eluted by subsequent washes (2 ml each) with elution buffer (low salt buffer containing 120 mM Imidazole). Fractions containing 55LCC were pooled and dialyzed for 4 h against 4 l of dialysis buffer [50 mM Tris pH 7.5, 300 mM NaCl, 5% (v/v) glycerol, 1 mM EGTA, 2 mM ADP, 2 mM MgCl_2_ and 1 mM tris(2-carboxyethyl)phosphine (TCEP)] at 4°C. The dialyzed sample was subsequently incubated with 3 ml Strep-Tactin Sepharose resin (IBA Lifesciences GmbH), pre-equilibrated in dialysis buffer, for 1 h at 4°C in rotation. After washing the resin four times with 50 ml of the same buffer, the 55LCC complex was eluted by subsequent washes (2 ml each) with elution buffer (dialysis buffer supplemented with 5 mM desthiobiotin). Fractions containing 55LCC were pooled, diluted three times in 25 mM Tris pH 7.5, 5% (v/v) glycerol, 1 mM EGTA, 2 mM ADP, 2 mM MgCl_2_ and 1 mM TCEP, and loaded onto a 5 ml HiTrap QHP column (Cytiva) pre-equilibrated in the same buffer. A linear 25 ml gradient, from 0.1 M to 1 M NaCl, was used to elute the complex. Fractions containing 55LCC were pooled, concentrated and resolved on a Superose 6 10/300 Increase column (Cytiva) pre-equilibrated in 25 mM Tris pH 7.5, 400 mM NaCl, 5% (v/v) glycerol, 1 mM EGTA, 2 mM ADP, 2 mM MgCl_2_ and 1 mM TCEP. Eluted peaks were analyzed by SDS-PAGE, and fractions containing >95% pure 55LCC or C1orf109-CINP were combined, concentrated to 1–2 mg/ml (for the four-subunits complex) or 4–6 mg/ml (for the C1orf109-CINP sub-complex), snap-frozen in liquid nitrogen, and stored at −80°C. Purification of 55LCC WB complex was carried out in an identical manner.

For native mass spectrometry experiments (see “Native mass spectrometry” section for details), the purified C1orf109-CINP sub-complex was incubated with 0.3 mg of 6xHis-TEV protease and dialyzed O/N against 4 l of dialysis buffer (low salt buffer containing 150 mM NaCl) at 4°C. Following removal of the TEV protease and uncleaved proteins by subtraction using 1 ml HisPur^™^ Ni-NTA resin (Thermo Fisher Scientific), the cleaved complex was concentrated and resolved on a Superdex 75 10/300 column (GE Healthcare) pre-equilibrated in 25 mM HEPES pH 7.5, 150 mM NaCl and 1 mM TCEP. Eluted peaks were analyzed by SDS-PAGE, and fractions containing >95% pure C1orf109-CINP were combined, concentrated to 6 mg/ml, snap-frozen in liquid nitrogen, and stored at −80°C.

#### Purification of SPATA5 variants

Purification of FL SPATA5 was carried out in a manner similar to the four-subunits complex, with the exception that the Strep-Tactin affinity step was omitted from the protocol. Following size-exclusion, the recovered protein was concentrated to 4–6 mg/ml, snap-frozen in liquid nitrogen, and stored at −80°C.

SPATA5 1–336 WT and mutant proteins were purified as described for 55LCC, with the exception that EGTA, ADP and MgCl_2_ were omitted from all buffers and 0.3 mg/ml lysozyme was added to the lysis buffer. Following nickel-affinity chromatography, fractions containing SPATA5 1–336 variants were pooled and dialyzed O/N against 4 l of dialysis buffer [50 mM Tris pH 7.5, 300 mM NaCl, 5% (v/v) glycerol and 1 mM TCEP] at 4°C. The dialyzed samples were subsequently diluted three times in 25 mM Tris pH 7.5, 5% (v/v) glycerol and 1 mM TCEP and loaded onto a 1 ml HiTrap QHP column (Cytiva) pre-equilibrated in the same buffer. A linear 25 ml gradient, from 0.1 M to 1 M NaCl, was used to elute each protein. Fractions containing SPATA5 1–336 WT and mutants were pooled, concentrated and resolved on a Superdex 75 10/300 column (GE Healthcare) pre-equilibrated in 25 mM Tris pH 7.5, 400 mM NaCl, 5% (v/v) glycerol and 1 mM TCEP. Eluted peaks were analyzed by SDS-PAGE, and fractions containing >95% pure SPATA5 1–336 variants were combined, concentrated to 0.6–7 mg/ml, snap-frozen in liquid nitrogen, and stored at −80°C.

#### Purification of CINP

CINP was purified using a protocol similar to the one described for 55LCC, with the exception that bacterial cells were resuspended in 100 ml ice-cold lysis buffer containing 50 mM Tris-HCl pH 7.6, 300 mM NaCl, 20 mM Imidazole, 5% (v/v) glycerol, 0.075% (v/v) β-mercaptoethanol, 1 mM benzamidine, 0.8 mM phenylmethylsulfonyl fluoride (PMSF) and 0.3 mg/ml lysozyme. Cells were lysed by sonication using a Sonics Vibracell instrument (1 s ON/3 s OFF, at 40% amplitude for 4 min), and the lysate was cleared by centrifugation at 30,000 g for 30 min at 4°C. The soluble fraction was then sonicated (1 s ON/3 s OFF, at 40% amplitude for 2 min) and subsequently passed through a 0.45 μm filter (Thermo Fisher Scientific). Filtered lysate was loaded onto a 5 ml HisTrap HP column (Cytiva), which was washed with four column volumes (CV) of low salt buffer [50 mM Tris-HCl pH 7.6, 300 mM NaCl, 20 mM Imidazole, 5% (v/v) glycerol, 0.075% (v/v) β-mercaptoethanol and 1 mM benzamidine], four CV of high salt buffer (low salt buffer containing 500 mM NaCl), and four CV of low salt buffer. A linear 20 CV gradient, from 20 mM to 300 mM Imidazole, was subsequently used to elute the protein. Eluted peaks were analyzed by SDS-PAGE, and fractions containing CINP were pooled and the 6xHis tag removed by addition of 0.3 mg 6xHis-TEV protease as described for the C1orf109-CINP complex. Following removal of the TEV protease and uncleaved 6xHis-CINP by subtraction using 1 ml HisPur^™^ Ni-NTA resin (Thermo Fisher Scientific), the cleaved protein was concentrated and resolved on a HiLoad Superdex 75 16/600 column (GE Healthcare) pre-equilibrated in 25 mM HEPES pH 7.5, 150 mM NaCl and 1 mM TCEP. Eluted peaks were analyzed by SDS-PAGE, and fractions containing >95% pure CINP were combined, concentrated to 5–10 mg/ml, snap-frozen in liquid nitrogen, and stored at −80°C.

#### Purification of C1orf109

C1orf109 was purified in a manner similar to CINP, with the exception that lysozyme was omitted from the lysis buffer and nickel-affinity chromatography was performed using HisPur^™^ Ni-NTA resin (Thermo Fisher Scientific) as described for 55LCC. Following removal of the TEV protease and uncleaved 6xHis-dStrepII-ybbR-C1orf109 by subtraction using 1 ml HisPur^™^ Ni-NTA resin (Thermo Fisher Scientific), the cleaved protein was concentrated and resolved on a Superdex 75 10/300 column (GE Healthcare) pre-equilibrated in 25 mM HEPES pH 7.5, 300 mM NaCl and 1 mM TCEP. Eluted peaks were analyzed by SDS-PAGE, and fractions containing >95% pure C1orf109 were combined, concentrated to ~0.3 mg/ml, snap-frozen in liquid nitrogen, and stored at −80°C.

#### Purification of VCP/p97

Purification of VCP/p97 was carried out in a manner similar to SPATA5, with the exception that 0.3 mg/ml lysozyme was added to the lysis buffer and the ion-exchange chromatography step was omitted from the protocol. Following nickel-affinity chromatography, the recovered protein was resolved on a HiLoad Superdex 200 16/600 column (GE Healthcare) pre-equilibrated in 25 mM Tris pH 7.5, 400 mM NaCl, 5% (v/v) glycerol, 1 mM EGTA, 2 mM ADP, 2 mM MgCl_2_ and 1 mM TCEP. Eluted peaks were analyzed by SDS-PAGE, and fractions containing >95% pure VCP/p97 were concentrated to 6–8 mg/ml, snap-frozen in liquid nitrogen, and stored at −80°C.

##### AlphaFold 3D structure prediction

To generate models for the monomeric forms of SPATA5, SPATA5L1, C1orf109, CINP and VCP/p97, the corresponding protein sequences were used as inputs in AlphaFold 2^33,34^. Structural predictions were run using the default settings on ColabFold^100^, asking for five models as outputs. AlphaFold-Multimer^35^ was used to obtain a model for the C1orf109-CINP complex, assuming a 1:1 stoichiometry. Protein sequences for C1orf109 and CINP were used as inputs, and five structural models were predicted using the default settings on ColabFold^100^.

##### Crystallization and structure determination of CINP

Crystals of FL CINP (10 mg/ml protein in 25 mM HEPES pH 7.5, 150 mM NaCl and 1 mM TCEP) were grown in Swissci 96-well 3-drop plates (Molecular Dimensions) using the vapor-diffusion technique at 19°C. Sitting drops were set up by combining 500 nl of purified protein with 500 nl of crystallization solution containing 25% (v/v) 1,2-propanediol, 10% (v/v) glycerol and 100 mM sodium potassium phosphate pH 6.2 (JBScreen JCSG++ HTS, Jena Bioscience), using a Mosquito crystal robot (Labtech). CINP crystals were harvested after one week and directly flash-cooled in liquid nitrogen without any additional cryoprotectant.

X-ray diffraction data were collected at 0.9795 Å, 100 K, on a Eiger2 XE 16M detector at the I04 beamline of Diamond Light Source (DLS). Data were auto-processed at DLS using a combination of autoPROC (for indexing, scaling, integrating and space group assignment)^101^ and Staraniso (for anisotropy determination and correction)^102^. The structure of CINP was solved by molecular replacement with the program Phaser-MR^103^ using the structure predicted by AlphaFold 2 as a search model. Structure refinement was performed with PHENIX v1.17.1^104^ using the TLS function available within the interface, while Coot v0.9.8.1^105^ was used to manually build missing residues. The overall quality of the model was assessed using MolProbity^106^. Data collection, refinement and validation statistics are summarized in Table S3b.

##### Cryo-electron microscopy – grids preparation and data collection

Prior to cryo-EM grids preparation, 55LCC (at 4.3 mg/ml) was incubated with 5 mM ATP***γ***S (Roche) at room temperature for 30 min. Quantifoil R3.5/1 200-mesh grids (Quantifoil Micro Tools GmbH) were glow-discharged for 30 s at 12 mA and 0.38 mBar pressure using a PELCO easiGlow system (Ted Pella). Fluorinated octyl maltoside (FOM; Molecular Dimensions) at a final concentration of 0.01% (v/v) was added to the ATP***γ***S-treated complex immediately before grids preparation, resulting in a complex concentration of 3.7 mg/ml. Cryo-EM grids were prepared by applying 3 μl of this sample onto the glow-discharged Quantifoil grids, followed by immediate blotting (blot force = 0 N, blot time = 8 s) and plunge-freezing in liquid ethane cooled by liquid nitrogen, using a FEI Vitrobot IV (Thermo Fisher Scientific) at 100% relative humidity and with a chamber temperature set at 4°C.

A dataset was collected on a FEI Titan KRIOS transmission electron microscope (Thermo Fisher Scientific) operating in counting mode at 300 keV, using a magnification of 165,000x and a pixel size of 0.74 Å. A total of 27,595 movies were recorded using the EPU automated acquisition software (v3.3) on a FEI Falcon 4i direct electron detector^107^ with an energy filter of 10 eV. A dose per physical pixel/s of 6.88 was used for each exposure, resulting in a total electron dose of 37.6 e^−^/Å^2^, fractionated across 918 EPU frames. These were then grouped into 20 frames, resulting in an electron dose of 0.8 e^−^/Å^2^ per frame. 20 exposures per hole were collected with an exposure time of 2.99 s each, defocus values ranging from −1.2 μm to −3.1 μm and an aberration free image shift (AFIS) range of 10 μm. Detailed information on data collection, refinement and validation statistics is shown in Table S3a.

##### Cryo-electron microscopy – data processing

A schematic of the data processing pipeline is shown in Figure S3B, and further details on the reported maps and model are available in Table S3a. Image processing was carried out using a combination of RELION v4.0^108^ and cryoSPARC v4.0.1^109^. Drift-corrected averages of each movie were created using RELION’s implementation of MotionCor2^110^, and real-time contrast transfer function (CTF) parameters of each determined using CTFFIND v4.1^111^. Motion correction and CTF estimation were carried out on-the-fly^107^. RELION template-based picking was employed for picking on all 27,595 movies, using a threshold of 0.52 and a minimum inter-particle distance of 120 Å. The resulting 4,182,380 picked particles were then extracted using a box size of 360 × 360 pixels and a binning factor of four, and subsequently subjected to iterative rounds of reference-free 2D classification with a mask diameter of 220 Å. After visual inspection, high quality 2D classes (486,495 particles) were re-extracted using a box size of 380 × 380 pixels without binning, and a previously determined 3D volume was also re-scaled to the same box and pixel sizes using “relion_image_handler”. The resulting particles and model were subjected to one round of 3D classification with C1 symmetry, which yielded two well-defined maps (234,500 total particles) representing the four-subunits 55LCC complex. These particles and the best-resolved model (Class 3; 24.8% data) were subjected to 3D refinement, per-particle CTF correction, particle polishing and again 3D refinement in RELION. The resulting refined model and particles were then imported into cryoSPARC and used to generate four initial 3D volumes, which were subsequently subjected to 3D classification using heterogeneous refinement with C1 symmetry. This yielded a well-resolved map (165,778 particles) that was used as a template for homogeneous and non-uniform 3D refinements with C2 symmetry, generating a map with a global resolution of 4.5 Å. To improve map quality, masks comprising the densities belonging to either the 55LCC lid or ATPase units were created using UCSF ChimeraX v1.6.1^112,113^. These focused masks, together with the particles and map obtained from non-uniform refinement, were used for local refinements with C2 symmetry. This generated improved maps with final resolutions of 4.02 Å and 4.52 Å for the lid and ATPase units respectively, which were used to assemble a composite map for the 55LCC complex. Final resolutions were determined using the gold-standard Fourier shell correlation criterion (FSC = 0.143); local resolutions were determined using the local resolution implementation in cryoSPARC.

##### Cryo-electron microscopy – model building and refinement

An atomic model for the 55LCC complex was generated by rigid body fitting AlphaFold 2 models of the individual subunits into the 55LCC composite map using UCSF ChimeraX v1.6.1^112,113^. The resulting model was then manually inspected and rebuilt using Coot v0.9.8.1^105^, and iterative rounds of real-space refinement were performed in Coot v0.9.8.1 and PHENIX v1.17.1^114^ using default parameters and secondary structure restraints. The SPATA5 and SPATA5L1 ATPase units and regions within their N-terminal domains displayed poor side chain density, and therefore the side chains of these regions were set to an occupancy of 0. Gaps were left where direct connectivity between secondary structure elements could not be determined. The overall quality of the model was assessed using MolProbity^106^.

##### Electrophoretic mobility shift assays

HPLC-purified DNA (Fw_1a and Fw_2–4) and RNA (Fw_1b) primers were purchased from Integrated DNA Technologies (IDT) and Sigma, respectively (refer to Table S6). Primers Fw_1a and Fw_1b were used as single-stranded (ss) DNA and RNA variants (72 nt each) respectively, while primers Fw_1a and Fw_2–4 were used to generate a replication fork (RF) DNA type (192 bp) as previously described^115^. Briefly, a 25 μl reaction was set up by mixing each primer at a final concentration of 36 μM in 70 mM Tris-HCl pH 7.5, 10 mM MgCl_2_ and 5 mM dithiothreitol (DTT). The tube was placed in a floating rack and then into a water bath set at 95°C. After 2 min, the water bath was switched off and allowed to slowly cool down O/N to room temperature. The integrity of assembled RF DNA was visualized by agarose gel electrophoresis, and quantified using a Nanodrop 8000 (Thermo Fisher Scientific). A Widom 601 double-stranded (ds) DNA fragment (175 bp) was kindly gifted by Dr. Marcus D. Wilson (University of Edinburgh).

DNA and RNA variants (2.2 ng/μl each corresponding to 84 nM, 17 nM and 16 nM of ss, ds and RF DNA respectively, and to 86 nM of RNA) were mixed with increased concentrations of 55LCC, SPATA5 or C1orf109-CINP (from 0.010 μM to 5.12 μM in 2-fold dilution steps) in EMSA buffer [15 mM Tris pH 7.5, 75 mM NaCl, 0.05 mg/ml BSA, 10% (v/v) glycerol, 0.05% (v/v) Triton X-100 and 1 mM DTT]. This resulted in protein:DNA ratio ranges of ~0.1–61, ~0.6–300 and ~0.6–319 for ss, ds and RF DNA variants, and of ~0.1–59 for RNA. Assays were incubated for 1 h on ice in a final volume of 10 μl, and reactions were stopped with the addition of 2 μl loading dye [EMSA buffer supplemented with 8% (w/v) sucrose and 0.01% (w/v) bromophenol blue]. Products were separated on a NativePAGE^™^ 3–12% Bis-Tris gel (Invitrogen), and run for 2–3 h at 100 V and 4°C in 0.5x Tris-Borate-EDTA (TBE) buffer. Gels were stained using Diamond^™^ nucleic acid dye (Promega) and visualized using an iBright 1500 gel imaging system (Thermo Fisher Scientific).

##### ATPase assays

ATPase assays were performed using the malachite green phosphate assay kit (MAK307; Sigma-Aldrich). Purified VCP/p97 (0.1 μM), SPATA5, 55LCC WT and WB (1 μM each) proteins were incubated in the absence or presence of RNA (AM7120G, Invitrogen; 2 μM) and of ss, ds and RF DNA (see “Electrophoretic mobility shift assays” section; 2 μM each) in assay buffer containing 25 mM HEPES pH 7.6, 100 mM NaCl, 1 mM ATP, 5% (v/v) glycerol, 10 mM MgCl_2_, 0.005% (v/v) Tween-20, 0.1 mg/ml BSA and 0.5 mM DTT. Enzyme reactions (60 μl final volume) were carried out in a MicroAmp™ TriFlex PCR reaction plate (Thermo Fisher Scientific), and incubated at 37°C for 150 min in a ProFlex PCR system (Life Technologies). To ensure the final ATP concentration was within the detectable range of the assay kit (< 0.25 mM), 4 μl samples were taken from each reaction at the indicated time points and mixed with 5 μl malachite green dye buffer (a mixture of reagents A and B as per manufacturer’s instructions) and 16 μl H_2_O in a 384-well white, μClear, flat-bottom non-binding plate (781903; Greiner bio-one). Following incubation for 30 min at room temperature, release of inorganic phosphate was measured by recording the absorbance at 620 nm in a Hidex Sense microplate reader. A reaction containing no protein was used to generate background readings of inorganic phosphate, which were subtracted from the experimental results at each time point. ATPase assays data were analyzed and plotted in GraphPad Prism 7 v7.0c (GraphPad Software).

##### Differential scanning fluorimetry

Purified SPATA5 1–336 WT and mutant proteins (~100–150 μl each) were dialyzed against 1 l assay buffer [25 mM HEPES pH 7.6, 200 mM NaCl, 5% (v/v) glycerol and 1 mM TCEP] O/N at 4°C prior to differential scanning fluorimetry measurements. Each protein and SYPRO^™^ Orange (Invitrogen) were then diluted in the same buffer to a final concentration of 4 μM and 5x respectively, in a total volume of 25 μl. Reactions were incubated at room temperature for 30 min before being analyzed on a QuantStudio 3 Real-Time PCR system (Thermo Fisher Scientific). The temperature was raised from 20°C to 95°C in 0.016°C/s intervals throughout the course of the assays. Data were analyzed using Protein Thermal Shift software v1.4 (Thermo Fisher Scientific) and plotted in GraphPad Prism 7 v7.0c (GraphPad Software).

##### Mass photometry

Microscope coverslides were prepared as described previously^116^. All mass photometry experiments were performed using a One^MP^ mass photometer instrument (Refeyn Ltd, Oxford, UK). The reconstituted 55LCC complex (at 3 μM), which was purified in the presence of 2 mM ADP (refer to “Protein purification” section for details), was incubated at room temperature for 30 min in a final volume of 10 μl prior to mass measurements. In a parallel reaction, the same complex (also at 3 μM) was mixed with 5 mM ATP***γ***S (Roche) and incubated at room temperature for 30 min in the same final volume. For each measurement, 16 μl of buffer [25 mM Tris pH 7.5, 400 mM NaCl, 5% (v/v) glycerol, 1 mM EGTA, 2 mM MgCl_2_ and 1 mM TCEP, supplemented with either 2 mM ADP or 5 mM ATP***γ***S depending on the reactions tested] was added to a well, and the focus point was found and adjusted as necessary. Protein mixtures were then diluted to 600 nM in their respective buffers and 4 μl of each dilution was added to the corresponding buffer droplet, resulting in final protein concentrations of 120 nM. Movies were recorded for 60 s using AcquireMP (Refeyn, Ltd, Oxford, UK), and processed using DiscoverMP (Refeyn, Ltd, Oxford, UK) as previously described^116^.

##### Native mass spectrometry

Untagged C1orf109, CINP and C1orf109-CINP (20 μl each at a concentration of 12 μM for C1orf109 and of 20 μM for both CINP and C1orf109-CINP complex) were buffer exchanged into 500 mM ammonium acetate using Zeba Spin 7K MWCO desalting columns (Thermo Fisher Scientific). Samples were analyzed by nanoelectrospray ionization mass spectrometry (MS) using a quadrupole-orbitrap MS (Q-Exactive UHMR, Thermo Fisher Scientific) and gold/palladium coated nanospray tips prepared in-house. The MS was operated in positive ion mode using a capillary voltage of 1.5 kV, capillary temperature of 250°C and S-lens RF of 200 V. In-source trapping was used with a desolvation voltage of −200 V for 4 ms. Extended trapping was not used. The quadrupole mass range was 2000–10000 m/z. Nitrogen gas was used in the HCD cell with a trap gas pressure setting of 5. Orbitrap resolution was 6250, detector *m/z* optimization was low. Five microscans were averaged and an AGC target of 2×105 was used. Mass calibration was performed by a separate injection of sodium iodide at a concentration of 2 mg/ml. Data processing was performed using QualBrowser v4.2.28.14 and deconvoluted using UniDec5^117^.

##### Cross-linking mass spectrometry

55LCC (50 μl at 4 μM) was dialyzed O/N against 1 l of 25 mM HEPES pH 7.5, 400 mM NaCl, 5% (v/v) glycerol, 1 mM EGTA, 2 mM ADP, 2 mM MgCl_2_ and 1 mM TCEP prior to cross-linking mass spectrometry experiments. The dialyzed complex was then incubated with 0.4 mM disuccinimidyl dibutyric urea (DSBU; Thermo Fisher Scientific) at room temperature for 30 min, and the reaction was quenched by adding 20 mM Tris pH 7.5. The proteins were digested using an S-Trap Micro (Protifi) according to the manufacturer’s instructions. Briefly, 25 μl of 10% (v/v) SDS was added to a 25 μl volume of cross-linked sample. DTT (220 mM in 50 mM ammonium bicarbonate pH 8.0, 5 μl) was then added and the mixture was incubated at 50°C for 10 min. Iodoacetamide (440 mM in 50 mM ammonium bicarbonate pH 8.0, 5.5 μl) was added and the mixture was incubated at 20°C for a further 30 min. The sample was acidified by adding phosphoric acid (55% phosphoric acid, 6 μl), followed by the addition of 0.1 M triethylammonium bicarbonate (TEAB) pH 7.1, 90% methanol (465 μl). Subsequently, trypsin was added (0.02 μg/μl in 100 mM TEAB) and the sample was loaded onto the S-Trap column. The column was washed with 0.1 M TEAB pH 7.1, 90% methanol (130 μl x 3) and trypsin solution was added (0.02 μg/μl trypsin in 100 mM TEAB pH 7.1, 30 μl). The digestion reaction was incubated for 1.5 h at 47°C. Peptides were eluted from the S-Trap in three sequential steps: (1) 50 mM TEAB (40 μl); (2) 0.2% formic acid (40 μl); (2) 50% acetonitrile, 0.2% formic acid (40 μl). The peptides were evaporated to dryness and resuspended in 2% acetonitrile, 0.1% formic acid (20 μl).

The peptides were then analyzed by liquid chromatography–mass spectrometry (LC–MS) on an Orbitrap Eclipse mass spectrometer interfaced with a Vanquish Neo liquid chromatography system (Thermo Fisher Scientific). Peptides (2 μl) were injected onto a 50 cm EASY-Spray column (Thermo Fisher Scientific) and separated by gradient elution of 2–35% (v/v) solvent B [0.1% (v/v) formic acid in acetonitrile] in solvent A [0.1% (v/v) formic acid in water] over 120 min at 300 nl min^−1^. The mass spectrometer was operated in data-dependent acquisition mode with precursor fragmentation performed by Higher-energy C-trap dissociation. Each high-resolution scan (m/z range = 380–1400, R = 60,000) was followed by product ion scans (R = 30,000), acquired using assisted collision energy mode with normalized collision energies of 25% and 30%. The cycle time was 3 s. Precursor ions with charge states 3–7+ (inclusive) were selected for tandem MS. The dynamic exclusion was set to 60 s. Cross-link identification was performed using MeroX 2.0.1.4^118^. Allowed cross-link sites were proteins N-termini, Lys, Tyr, Ser, Thr. A maximum of two of the four marker ions, corresponding to fragmentation of the cross-linker, were allowed to be missing in the database search. The MS1 and MS2 error tolerances were set to 8 and 20 ppm, respectively and up to three missed cleavages were allowed. The false discovery rate was set to 1%. xiView^119^ was used to visualize cross-linking results.

##### Structural visualization and sequence analysis

All structural representations depicted in Figures 3B, S2A, and S4A were created in PyMOL (The PyMOL Molecular Graphic System, v2.2.3 Schrödinger, LLC). Structural models and surface representations shown in Figures 2D, 3A, 4A, S3B and D–I, S4B, and Movie S1 were obtained using UCSF Chimera X v1.6.1^112,113^. Superimpositions of the SPATA5, SPATA5L1 and VCP/p97 models shown in Figure S2A, and of the crystal structure and AlphaFold 2 models from CINP and C1orf109 shown in Figure S4A, were performed using the program Superpose^120^, available within the CCP4 suite (Collaborative Computational Project, Number 4, 1994). The 3D FSC plots shown in Figures S3D–F were generated using the remote 3DFSC processing server, available at https://3dfsc.salk.edu^121^. Sequence alignments shown in Figures S2B, S3H, and S4C and D were performed with MUSCLE^93,94^, and edited and displayed using Aline v1.0.025^122^. The final model depicted in Figure 7E was created with BioRender (https://biorender.com/).

### Quantification and statistical analysis

Statistical analyses and graphs were generated using GraphPad Prism 10 and Microsoft Excel. Generally, statistical significance was determined by unpaired Student’s *t*-test or one-way ANOVA with Tukey’s multiple comparison test. Details of the statistical analyses performed, resulting *p*-values, and number of replicates are mentioned in the figure legends. Error bars represent SEM (Standard Error of Mean) and ‘n’ generally refers to number of replicates in most experiments except in Figures 5C and D where n = number of fibers and Figures 5E, F, and S5E where n = number of cells. n.s. = not significant, * = p value ≤0.05, ** = p value ≤0.01, *** = p value ≤0.001, **** = p value ≤0.0001.

## Supplementary Material

supporting figures

## Data Availability

The atomic coordinates and structure factors for FL CINP can be accessed from the Protein Data Bank under the accession code PDBid: 8CIH. The cryo-EM maps and model for the 55LCC complex can be accessed from the Electron Microscopy Data Bank and the Protein Data Bank under the accession codes EMD-19177 and PDBid: 8RHN. Raw XL-MS data have been deposited to the ProteomeXchange Consortium via the PRIDE partner repository with the dataset identifier PXD035980 (Username: reviewer_pxd035980@ebi.ac.uk; Password: k88VS08j). All IP-mass spectrometry data are available in Table S1. Raw data from Figures 1, 4–7, S1, S4–S7 were deposited on Mendeley at DOI: 10.17632/35jh7n33gm.1. All deposited data will be made publicly accessible upon publication. This paper does not report any original code. Any additional information required to reanalyze the data reported in this paper is available from the lead contact upon request.

## Highlights

- SPATA5 and SPATA5L1 form a hetero-hexameric complex with C1orf109-CINP (55LCC)
- 55LCC associates with chromatin and exhibits DNA stimulated ATPase activity
- 55LCC loss causes proteotoxicity, replication stress, and genome instability
- 55LCC processes replisome substrates in response to replication fork insults

## In Brief

The 55LCC heterohexameric ATPase complex promotes replisome proteostasis to maintain replication fork progression and genome stability.
