# Supplementary material for: The SPATA5-SPATA5L1 ATPase complex directs replisome proteostasis to ensure genome integrity
Source: Cell. Author manuscript; Available in PMC 2025 Jul 31. (PMC11055677; doi:10.1016/j.cell.2024.03.002)
Supplement: supporting figures [file EMS206816-supplement-supporting_figures.pdf]

Anton Paper 11 supplements

2024 Apr 25;187(9):2250-2268.e31.

doi: 10.1016/j.cell.2024.03.002. Epub 2024 Mar 29.

The SPATA5-SPATA5L1 ATPase complex directs replisome proteostasis to ensure genome integrity

Vidhya Krishnamoorthy [1], Martina Foglizzo [2], Robert L Dilley [3], Angela Wu [1], Arindam Datta [1], Parul Dutta [1], Lisa J Campbell [2], Oksana Degtjarik [2], Laura J Musgrove [2], Antonio N Calabrese [2], Elton Zeqiraj [4], Roger A Greenberg [5]

Abstract

Ubiquitin-dependent unfolding of the CMG helicase by VCP/p97 is required to terminate DNA replication. Other replisome components are not processed in the same fashion, suggesting that additional mechanisms underlie replication protein turnover. Here, we identify replisome factor interactions with a protein complex composed of AAA+ ATPases SPATA5-SPATA5L1 together with heterodimeric partners C1orf109-CINP (55LCC). An integrative structural biology approach revealed a molecular architecture of SPATA5-SPATA5L1 N-terminal domains interacting with C1orf109-CINP to form a funnel-like structure above a cylindrically shaped ATPase motor. Deficiency in the 55LCC complex elicited ubiquitin-independent proteotoxicity, replication stress, and severe chromosome instability. 55LCC showed ATPase activity that was specifically enhanced by replication fork DNA and was coupled to cysteine protease-dependent cleavage of replisome substrates in response to replication fork damage. These findings define 55LCC-mediated proteostasis as critical for replication fork progression and genome stability and provide a rationale for pathogenic variants seen in associated human neurodevelopmental disorders.

Figures

Figure 1. SPATA5 and SPATA5L1 form a complex with CINP and C1orf109 that interacts with the POLD3 replisome and is essential for cell viability and protein homeostasis.

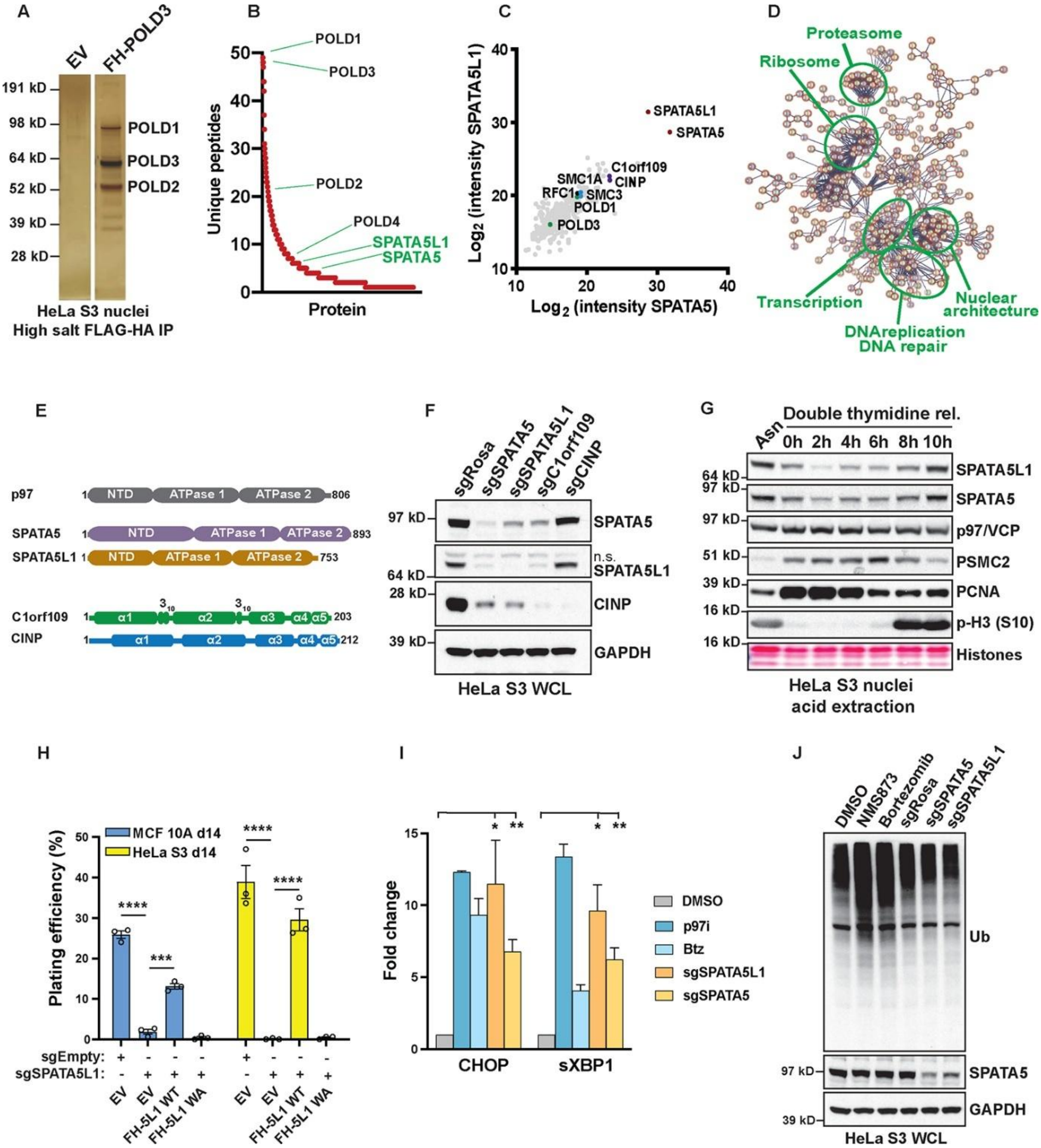

A. Silver stain of FLAG-HA tandem purified samples from high-salt extracted HeLa S3 nuclei stably expressing FH-POLD3 or EV. TCA precipitated samples were analyzed by mass spectrometry. Data in Table S1. FH = FLAG-HA, EV = empty vector.

B. Quantification of mass spectrometry unique peptide number for proteins identified in A.

C. Mass spectrometry of FLAG-HA affinity purified samples from high-salt extracted HeLa S3 nuclei stably expressing FH constructs or EV with data analysis represented in C-D. Quantification of the intensities of the identified proteins from FH-SPATA5 and FH-SPATA5L1. Data in Table S1.

D. STRING interaction network of proteins from mass spectrometry of FH-SPATA5L1 purified sample.

E. Schematics depicting the domain organization of indicated proteins. NTD = N-terminal domain.

F. Western blot of whole cell lysates (WCL) from HeLa S3 treated with indicated CRISPR guides (sg) or control (sgRosa) for 8 days. n.s. indicates non-specific bands.

G. Western blot of acid extracted nuclei from asynchronous (Asn) and double thymidine block released samples of HeLa S3. Histones visualized with Ponceau stain.

H. Quantification of plating efficiency of MCF 10A and HeLa S3 stably expressing FHS-PATA5L1 constructs or EV treated with indicated CRISPR guides for 14 days. Data are mean α SEM; n=3 from 1 biological replicate. *p*-values determined by unpaired Student's *t*-test. WT = wild type, WA = Walker A double mutant.

I. RealTime-qPCR of integrated stress response genes CHOP and spliced XBP1 of HeLa S3 cells treated with indicated inhibitors for 4 hours (h) or CRISPR guides for 10 days. n=3 biological replicates; n=2 for Bortezomib (Btz) and sgSPATA5 samples. *p*-values determined by unpaired Student's *t*-test.

J. Western blot of WCL from HeLa S3 treated with inhibitors for 4 h or the indicated CRISPR guides for 8 days.

Figure 2. The SPATA5-SPATA5L1-C1orf109-CINP (55LCC) complex is a DNA-binding ATPase.

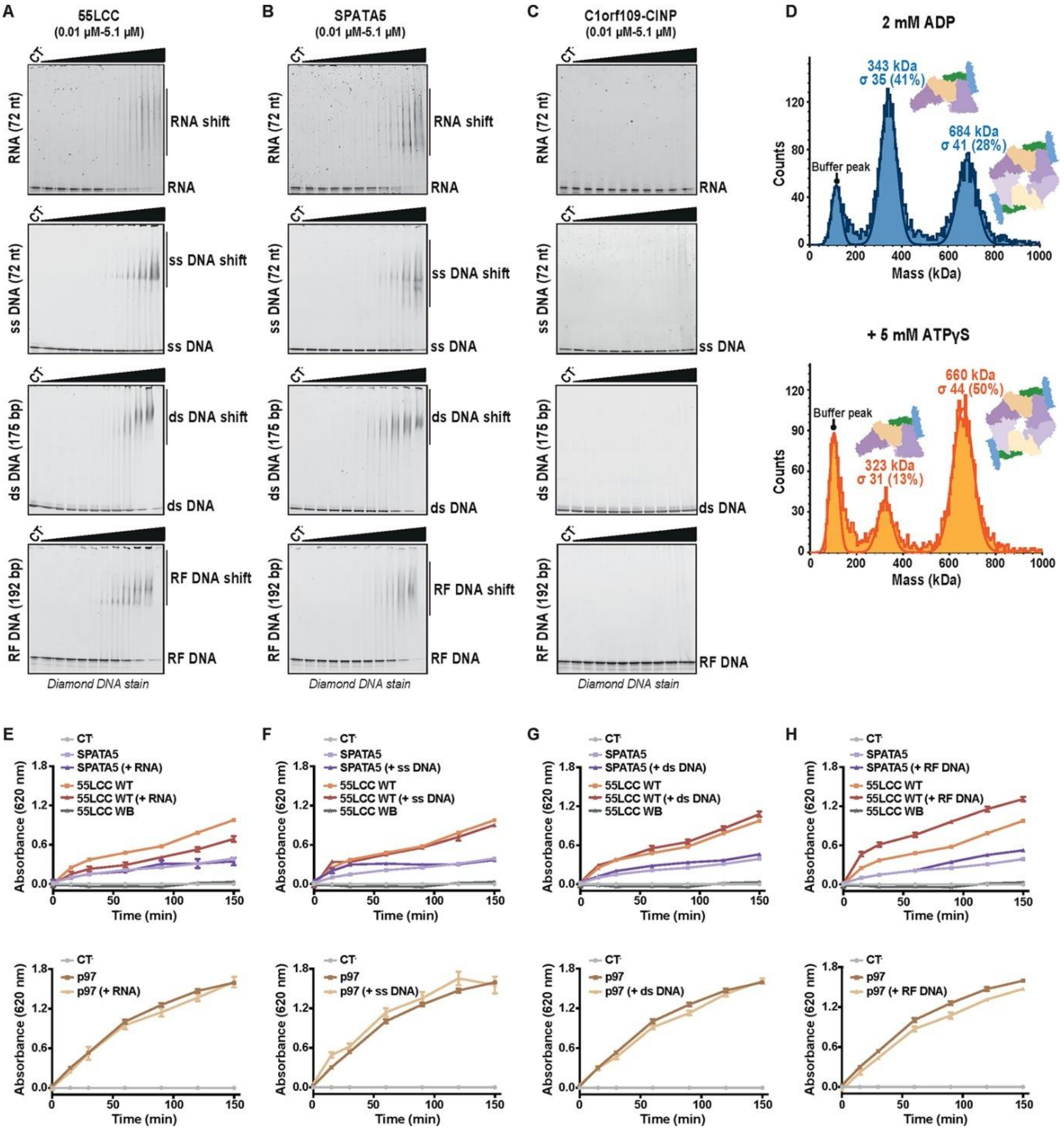

A.-C. Electrophoretic mobility shift assays (EMSAs) testing 55LCC (**A**), SPATA5 (**B**), and C1orf109-CINP (**C**) binding to RNA and to single-stranded (ss), double-stranded (ds) and replication fork (RF) DNA variants (2.2 ng/μl each corresponding to 84 nM, 17 nM and 16 nM of ss, ds and RF DNA respectively, and to 86 nM of RNA) at increasing protein concentrations (from 0.01 μM to 5.1 μM in 2-fold dilution steps). This resulted in protein:DNA ratio ranges of ~0.1–61, ~0.6–300 and ~0.6–319 for ss, ds and RF DNA variants, and of ~0.1–59 for RNA. Gels were stained with Diamond™ nucleic acid dye. Data are representative of two independent experiments. CT⁻ = negative control.

D. Mass distribution of 55LCC measured by mass photometry in the presence of 2 mM ADP (*top*), and after addition of 5 mM ATPγS (*bottom*). Observed masses for each peak are indicated. The percentage of particles contributing to each peak is shown in brackets (see Table S2 for details). Schematics depicting indicated complexes and presumed sub-complexes consistent with the measured Mw are shown; SPATA5 and SPATA5L1 subunits are colored in shades of purple and orange respectively, while C1orf109-CINP are colored green and dark blue. Data are representative of two independent experiments carried out in technical duplicates.

E.-H. ATPase activities of SPATA5, 55LCC variants (at 1 μM each; *top*) and p97 (at 0.1 μM; *bottom*) were determined in the absence or presence of RNA (**E**), and of ss (**F**), ds (**G**) and RF (**H**) DNA types (at 2 μM each). Results are the average of three independent experiments carried out in technical duplicates. Mean with SEM was used to plot the data; error bars shorter than the height of the symbol are not visible. CT⁻ = negative control, WT = wild-type, WB = Walker B double mutant.

Figure 3. Analyses of 55LCC and C1orf109-CINP complexes.

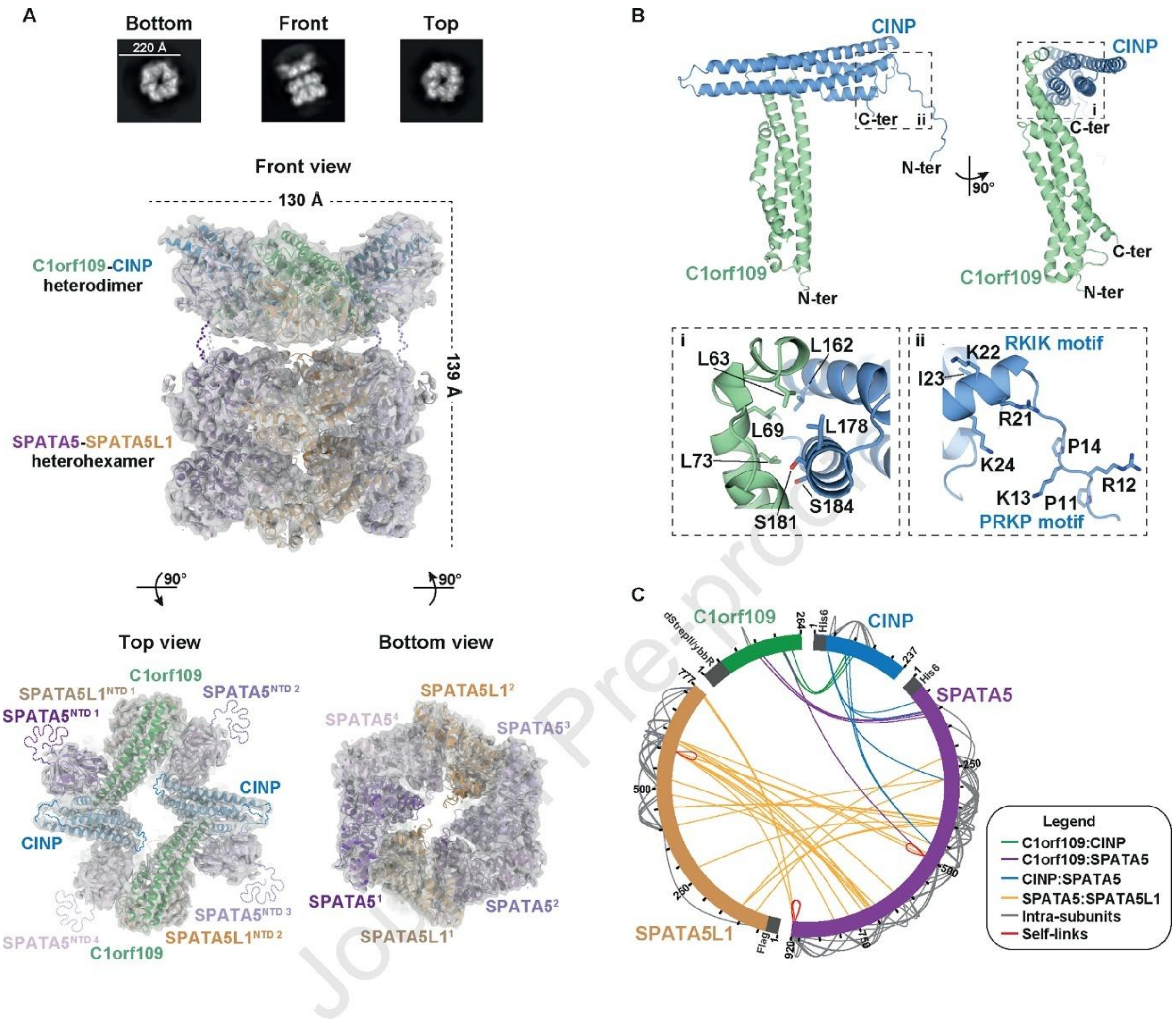

Journal Pre-proof

A. Representative 2D class averages from cryo-EM analysis of 55LCC. Data are representative of 165,778 selected particles (*top*). Cryo-EM composite map of 55LCC at contour level of 0.131 is depicted in transparent surface, with front, top and bottom views indicated. Cartoon models of the C1orf109-CINP heterodimer and the SPATA5-SPATA5L1 heterohexamer were rigid-body fitted in the cryo-EM density and colored as in Figure 2D. Unresolved linker regions connecting the 55LCC lid and ATPase domains, missing loops within each CINP promoter or unstructured regions in the SPATA5 N-termini are indicated with dashed lines; superscript numbers refer to the SPATA5 and SPATA5L1 subunits (*bottom*). See Figures S3B–H and Table S3a for details. NTD = N-terminal domain.

B. Cartoon model of the C1orf109-CINP complex predicted by AlphaFold-Multimer, with C1orf109 and CINP colored in green and dark blue respectively; dashed black rectangles highlight regions analyzed by mutagenesis (*top*). Close-up views and structural details of the predicted interacting residues at the C1orf109-CINP interface (panel i), and of CINP RKIK and PRKP motifs (panel ii). Residues are labeled and shown as sticks (*bottom*).

C. Chemical cross-linking and mass spectrometry analyses of the reconstituted 55LCC complex. Purification and labeling tags are indicated and colored gray. Data are representative of a single experiment measured in duplicate.

Figure 4. Analysis of patient mutations in SPATA5 and SPATA5L1.

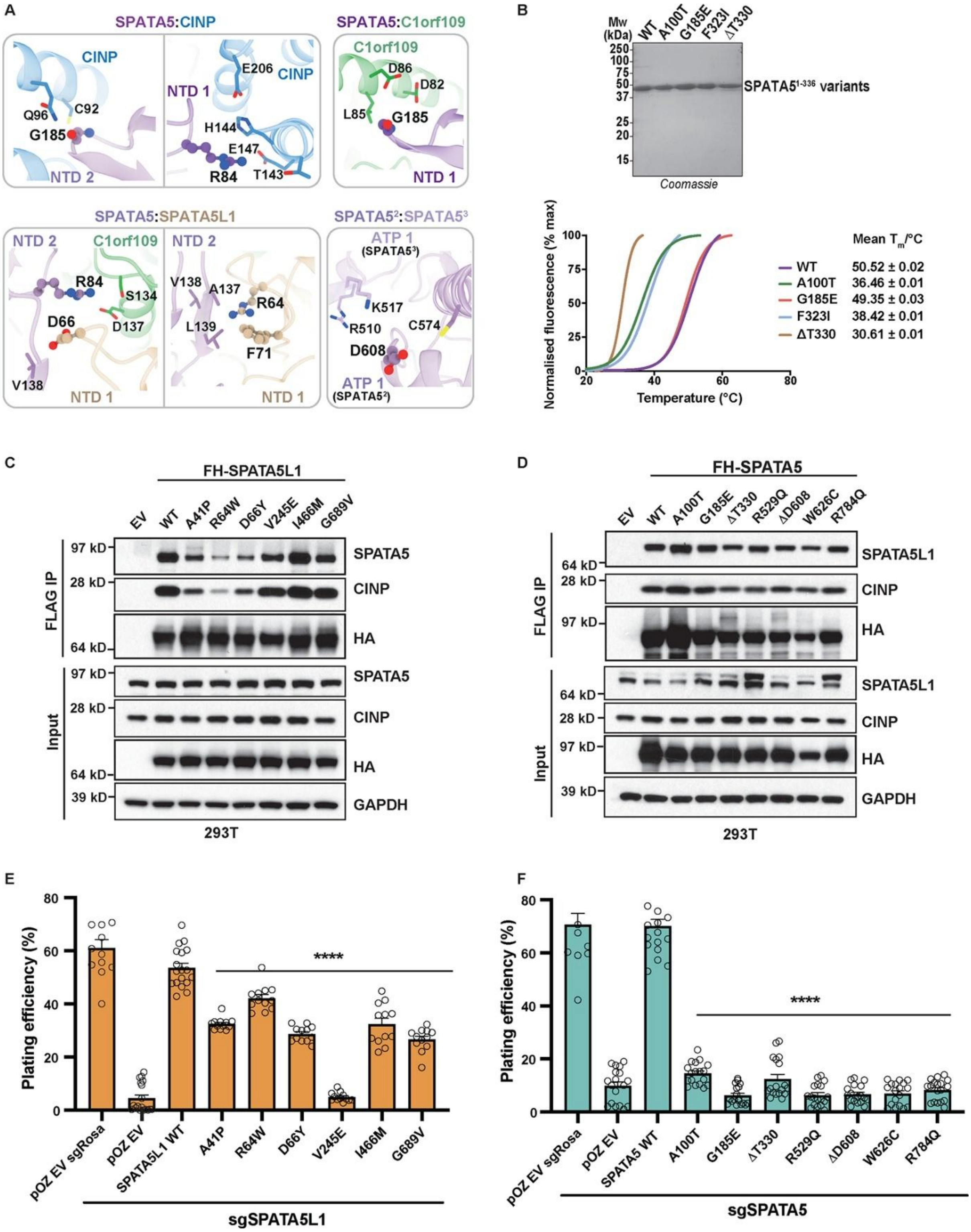

A. Close-up views and structural details of SPATA5 and SPATA5L1 residues mutated in neurodevelopmental syndromes. 55LCC subunits are shown in cartoon representation and colored as in Figure 2D. Mutated residues are labeled and shown as ball & sticks, while surrounding amino acids are indicated and shown as sticks. Superscript numbers

refer to the SPATA5 subunits. See Figure S2B, Table S4, and Movie S1 for details. NTD = N-terminal domain, ATP 1 = ATPase 1 domain.

B. Coomassie-stained SDS-PAGE analysis of purified SPATA5$^{1-336}$ WT and disease mutants. Data are representative of a single experiment (*top*). Differential scanning fluorimetry analysis of SPATA5$^{1-336}$ WT and disease variants. All proteins were assayed at 4 μM, and the calculated mean melting temperature (T_m) values for each are shown. Results are the average of three independent experiments carried out in technical duplicates. Mean with SEM was used to calculate the T_m values for each protein (*bottom*). WT = wild type.

C. Western blot of FLAG IP and input samples from 293T transiently expressing FH-SPATA5L1 patient mutations or EV. n=2 biological replicates.

D. Western blot of FLAG IP and input samples from 293T transiently expressing FH-SPATA5 patient mutations or EV. n=2 biological replicates.

E. Quantification of plating efficiency of HeLa S3 stably expressing FH-SPATA5L1 patient mutants or EV treated with indicated CRISPR guides for 14 days. n=12 from 2 biological replicates. SPATA5L1 WT and pOZ EV sgSPATA5L1 data are from n=18 from 3 biological replicates. Data are presented as mean ± SEM. *p*-values determined by one-way ANOVA with Tukey's multiple comparison test.

F. Quantification of plating efficiency of HeLa S3 stably expressing FH-SPATA5 patient mutants or EV treated with indicated CRISPR guides for 14 days. n=18 from 3 biological replicates. pOZ EV sgRosa data are from n=12 from 2 biological replicates. Data are presented as mean ± SEM. *p*-values determined by one-way ANOVA with Tukey's multiple comparison test.

Figure 5. 55LCC is required for DNA replication and genome stability.

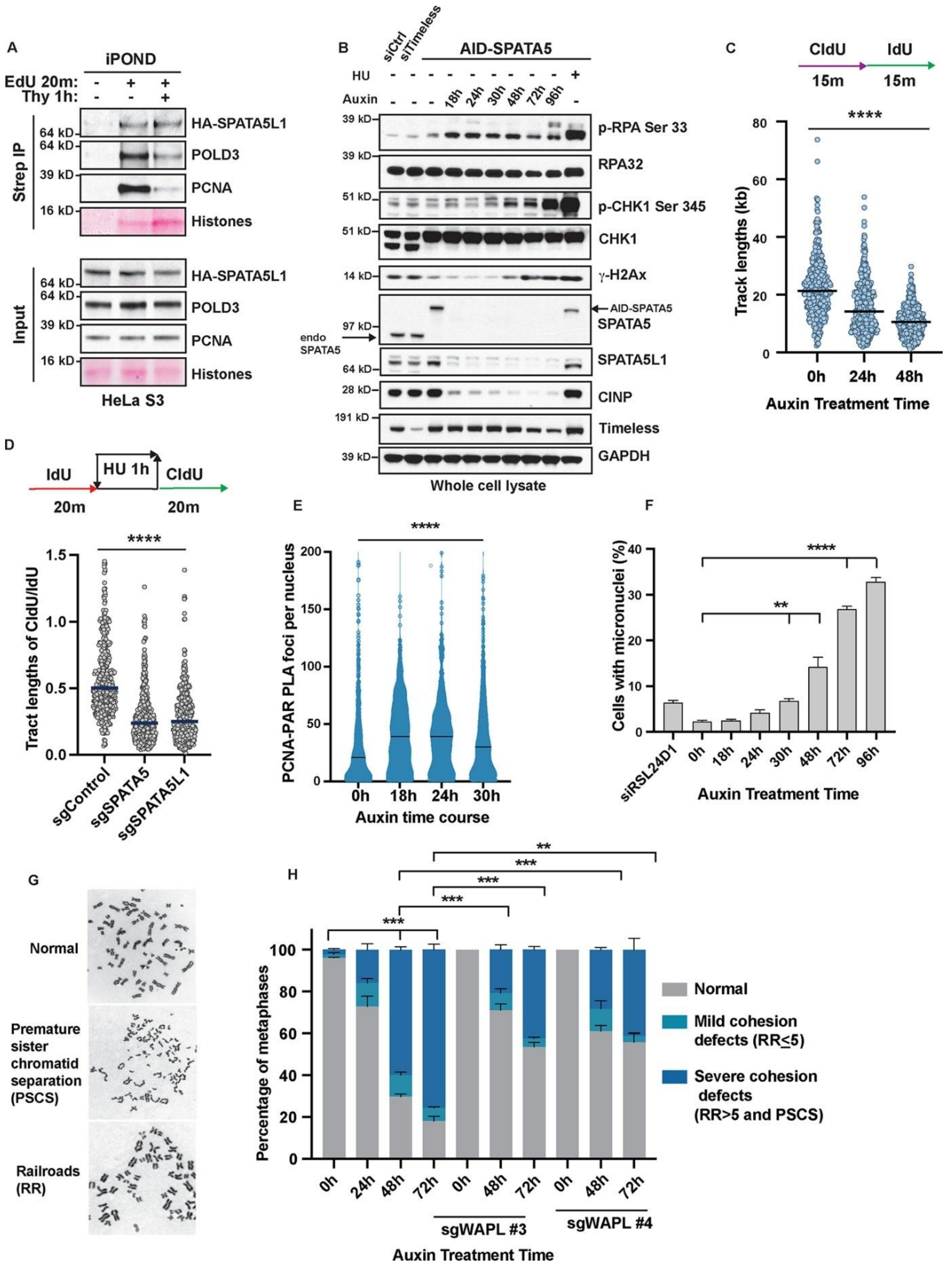

A. Western blot of Streptavidin IP and input of iPOND experiment from HeLa S3 stably expressing FH-SPATA5L1 treated −/+ EdU and −/+ Thymidine chase (Thy) for indicated times. n=2 biological replicates.

B. Western blot of WCL from HeLa S3 AID-SPATA5 cells treated with auxin for indicated times. HeLa S3 cells treated with indicated siRNAs for 72 h and AID-SPATA5 cells treated with 1 mM HU for 20 h were used as additional controls. n=3 biological replicates.

C. Schematic and quantification of individual DNA fiber track lengths in HeLa S3 AID-SPATA5 cells treated with auxin for indicated times. Median is indicated. *p*-value determined by one-way ANOVA with Tukey's multiple comparison test. N≥500 fibers sampled over three independent experiments.

D. Schematic and quantification of replication fork restart assay in HeLa S3 cells treated with the indicated CRISPR guides for 8 days. Median is indicated. *p*-value determined by one-way ANOVA with Tukey's multiple comparison test from n≥400 fibers sampled over three independent experiments.

E. Quantification of proximity ligation assay (PLA) of PCNA and ADP-Ribose in HeLa S3 AID-SPATA5 cells treated with auxin for indicated times. PLA foci per nucleus are plotted and median is indicated. n≥400 cells sampled over 3 biological replicates. *p*-value determined by one-way ANOVA with Tukey's multiple comparison test.

F. Quantification of DAPI-stained HeLa S3 AID-SPATA5 cells treated with auxin for indicated times. HeLa S3 cells treated with indicated siRNAs for 72 h were used as additional controls. Data are presented as mean ± SEM, n≥3000 cells sampled over 3 biological replicates. *p*-value determined by unpaired Student's *t*-test.

G.-H. Representative images (**G**) and quantification (**H**) of cohesion defects upon auxin treatment in HeLa S3 AID-SPATA5 cells treated with indicated CRISPR guides for WAPL. Data are presented as mean ± SEM from 3 biological replicates and at least 50 metaphase spreads analyzed per sample per experiment. *p*-values derived from unpaired Student's *t*-test for severe cohesion defects.

Figure 6. 55LCC mediates cleavage of replication factors.

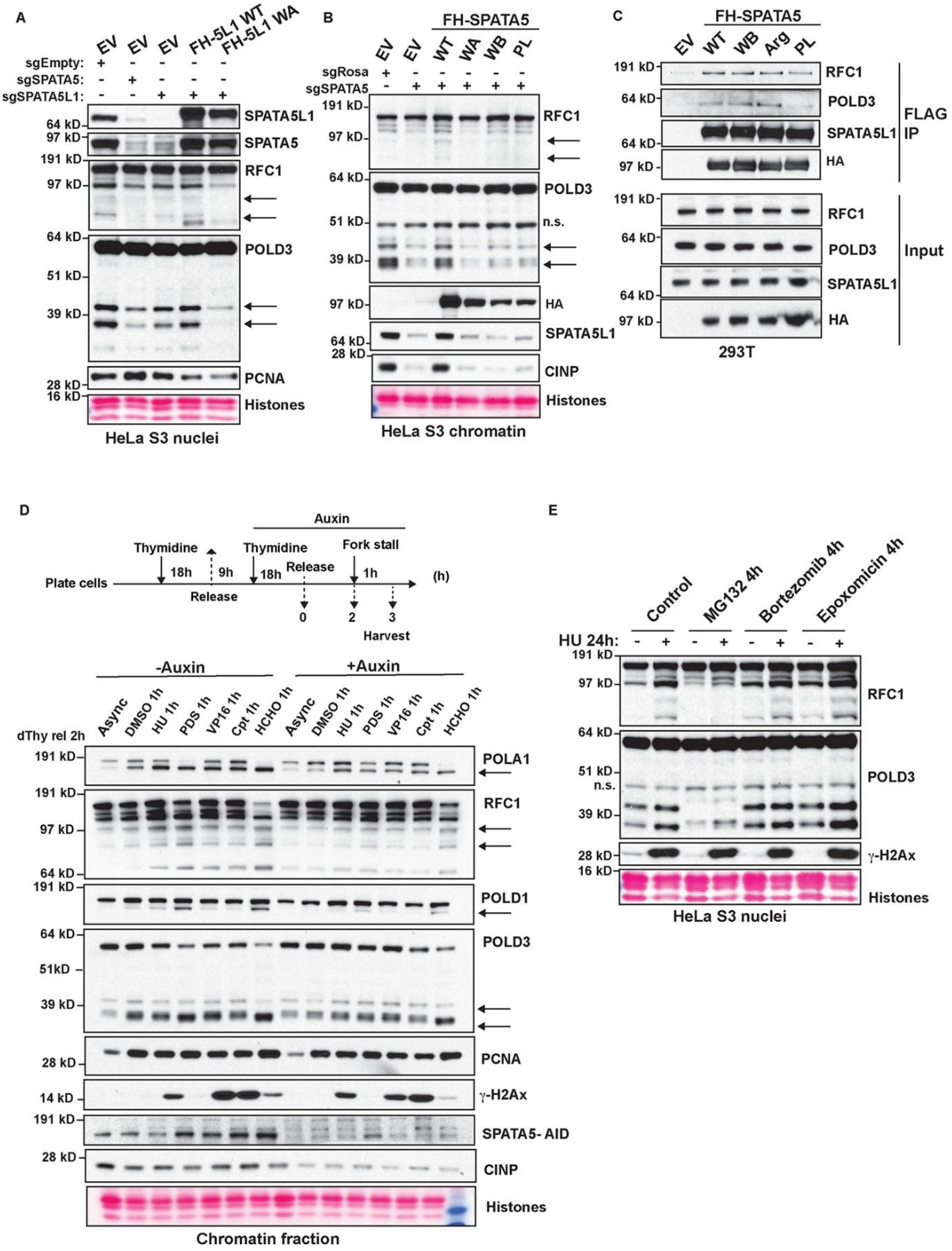

A. Western blot of acid extracted nuclei from HeLa S3 stably expressing FH-SPATA5L1 constructs or EV and treated with indicated CRISPR guides for 7 days. n=3 biological replicates. Arrows indicate cleaved products. Quantification and graphs in Table S5.

B. Western blot of acid extracted chromatin from HeLa S3 cells stably expressing FH-SPATA5 constructs treated with the indicated guides for 8 days. WB = Walker B double mutant, Arg = Arginine finger double mutant, PL = Pore loop double mutant. n=3 biological replicates. n.s. indicates non-specific bands. Arrows indicate cleaved products. Quantification and graphs in Table S5.

C. Western blot of FLAG IP and input samples from 293T cells transiently expressing FH-SPATA5 constructs or EV. n=3 biological replicates.

D. Schematic and western blot of acid extracted chromatin from HeLa S3 AID-SPATA5 cells treated with −/+ Auxin for indicated time, released from double thymidine (dThy) for 2 h and treated with indicated drugs for 1 h. Async = Asynchronous. n=3 biological replicates. Arrows indicate cleaved products. Quantification and graphs in Table S5.

E. Western blot of acid extracted nuclei from HeLa S3 treated with inhibitors for indicated times. n=3 biological replicates.

Figure 7. Cysteine protease-dependent proteolysis of the replisome.

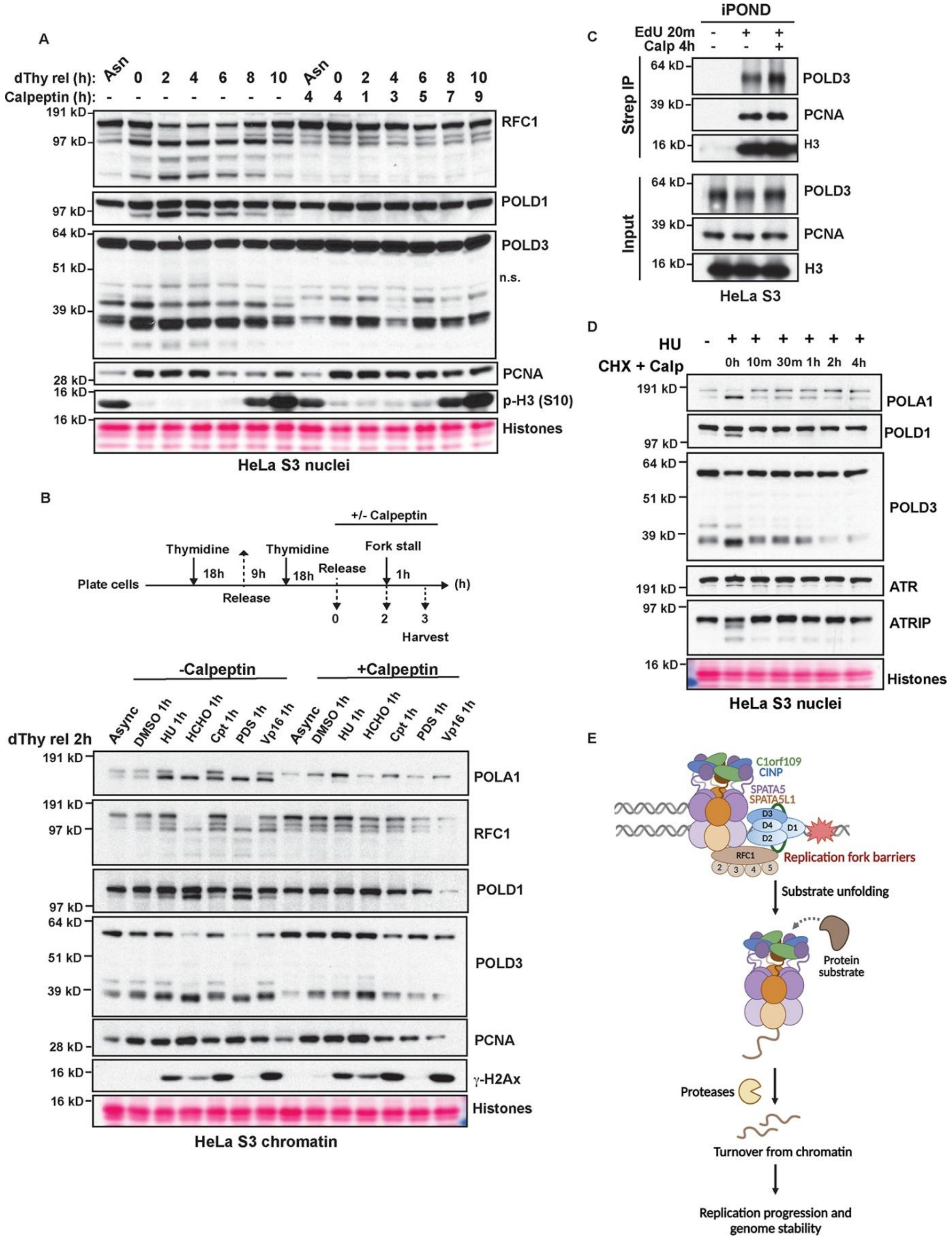

A. Western blot of acid extracted nuclei from HeLa S3 released from double thymidine (dThy) and treated −/+ Calpeptin for indicated times. n.s. indicates non-specific bands. Asn = Asynchronous. n=3 biological replicates. Mean relative signal intensities of POLD3 and RFC1 full length proteins normalized to histones are quantified in Table S5.

B. Schematic and western blot of acid extracted chromatin from HeLa S3 cells released from double thymidine (dThy) −/+ Calpeptin for 2 h and treated with indicated drugs for 1h. n=3 biological replicates. Async = Asynchronous.

C. Western blot of Streptavidin IP and input of iPOND experiment from HeLa S3 cells treated with −/+ Calpeptin followed by −/+ EdU for indicated times. n=2 biological replicates.

D. Western blot of acid extracted nuclei from HeLa S3 +/− 2 mM HU for 24 h followed by treatment with Calpeptin + Cycloheximide (CHX) for indicated times. n=3 biological replicates.

E. Model depicting the function of 55LCC in unfolding and proteolysis of replisome components, thus maintaining genome stability.
